# Genomic impact of severe population decline in a nomadic songbird

**DOI:** 10.1371/journal.pone.0223953

**Published:** 2019-10-24

**Authors:** Ross Crates, George Olah, Marcin Adamski, Nicola Aitken, Sam Banks, Dean Ingwersen, Louis Ranjard, Laura Rayner, Dejan Stojanovic, Tomasz Suchan, Brenton von Takach Dukai, Robert Heinsohn

**Affiliations:** 1 Fenner School of Environment and Society, Australian National University, Canberra, ACT, Australia; 2 Research School of Biology, Australian National University, Canberra, ACT, Australia; 3 BirdLife Australia, Carlton, Melbourne, VIC, Australia; 4 W. Szafer institute of Botany, Polish Academy of Sciences, Krakow, Poland; University of British Columbia Okanagan, CANADA

## Abstract

Uncovering the population genetic histories of non-model organisms is increasingly possible through advances in next generation sequencing and DNA sampling of museum specimens. This new information can inform conservation of threatened species, particularly those for which historical and contemporary population data are unavailable or challenging to obtain. The critically endangered, nomadic regent honeyeater *Anthochaera phrygia* was abundant and widespread throughout south-eastern Australia prior to a rapid population decline and range contraction since the 1970s. A current estimated population of 250–400 individuals is distributed sparsely across 600,000 km^2^ from northern Victoria to southern Queensland. Using hybridization RAD (hyRAD) techniques, we obtained a SNP dataset from 64 museum specimens (date 1879–1960), 102 ‘recent’ (1989–2012) and 52 ‘current’ (2015–2016) wild birds sampled throughout the historical and contemporary range. We aimed to estimate population genetic structure, genetic diversity and population size of the regent honeyeater prior to its rapid decline. We then assessed the impact of the decline on recent and current population size, structure and genetic diversity. Museum sampling showed population structure in regent honeyeaters was historically low, which remains the case despite a severe fragmentation of the breeding range. Population decline has led to minimal loss of genetic diversity since the 1980’s. Capacity to quantify the overall magnitude of both genetic diversity loss and population decline was limited by the poorer quality of genomic data derived from museum specimens. A rapid population decline, coupled with the regent honeyeater’s high mobility, means a detectable genomic impact of this decline has not yet manifested. Extinction may occur in this nomadic species before a detectable genomic impact of small population size is realised. We discuss the implications for genetic management of endangered mobile species and enhancing the value of museum specimens in population genomic studies.

## Introduction

Large-scale habitat loss and fragmentation is a widespread and ongoing global process [1], affecting the demographics and population genetics of species through altered patterns of dispersal, reproduction and selection [2]. Demographic and genetic effects of anthropogenic habitat loss differ substantially between species, depending upon life-history traits [3]. Species with limited dispersal capacity and short generation times are particularly susceptible to rapid loss of genetic variation within populations, increases in genetic structure among populations and reduced fitness, following habitat fragmentation [4]. In contrast, highly mobile species are typically, but not universally [5], less susceptible to negative genetic effects of habitat fragmentation, through their capacity to maintain gene flow over larger distances [6, 7].

While mobile species may tolerate some structural fragmentation of habitat before population processes are impacted, they often depend upon a spatial network of habitats over time. Loss or degradation of critical habitat elements is a major risk [8]. Alternative population pressures such as harvesting can have also major negative impacts [9, 10]. In the case that functional population connectivity is reduced, mobile species may be particularly susceptible to rapid population decline. Negative genetic effects such as inbreeding depression can emerge once populations become small or fragmented [4], as rapid population decline limits opportunities to purge deleterious alleles from the population [11].

For rare and highly mobile species, quantifying the impacts of habitat loss on demographics and population genetics is challenging [12, 7]. Limited spatio-temporal sampling can hinder detection of the genetic effects of population decline, which may manifest before or after sampling windows [13, 14]. Similarly, obtaining sufficient samples to detect recent changes to the size and genetic composition of small and sparsely-distributed populations can be difficult [15, 16]. When possible, however, understanding historical demographics can help quantify anthropogenic impacts on population size [9]. Because contemporary population genetics are the result of past demographic processes, combining genetic information from historical and contemporary samples can also assist conservation efforts [17, 18]. Such information can delineate conservation units [7, 19, 20], aid genetic management of captive populations [13], identify priority areas for habitat restoration or translocation [21], and quantify genetic threats in high risk species [14].

Continuing developments in high-throughput sequencing offers increasingly greater resolution to uncover the genetic effects of population decline, despite small sample sizes [22]. Capacity to obtain single nucleotide polymorphism (SNP) data from ancient (a)DNA sources including museum specimens offers exciting opportunities to infer the historical demographics and population genomics of threatened species, prior to their decline [12, 23]. Yet for non-model organisms, the type of information that can be inferred about past population processes using museum SNP data, along with the most robust techniques for doing so, is subject to ongoing research [14, 24].

**Fig 1:**
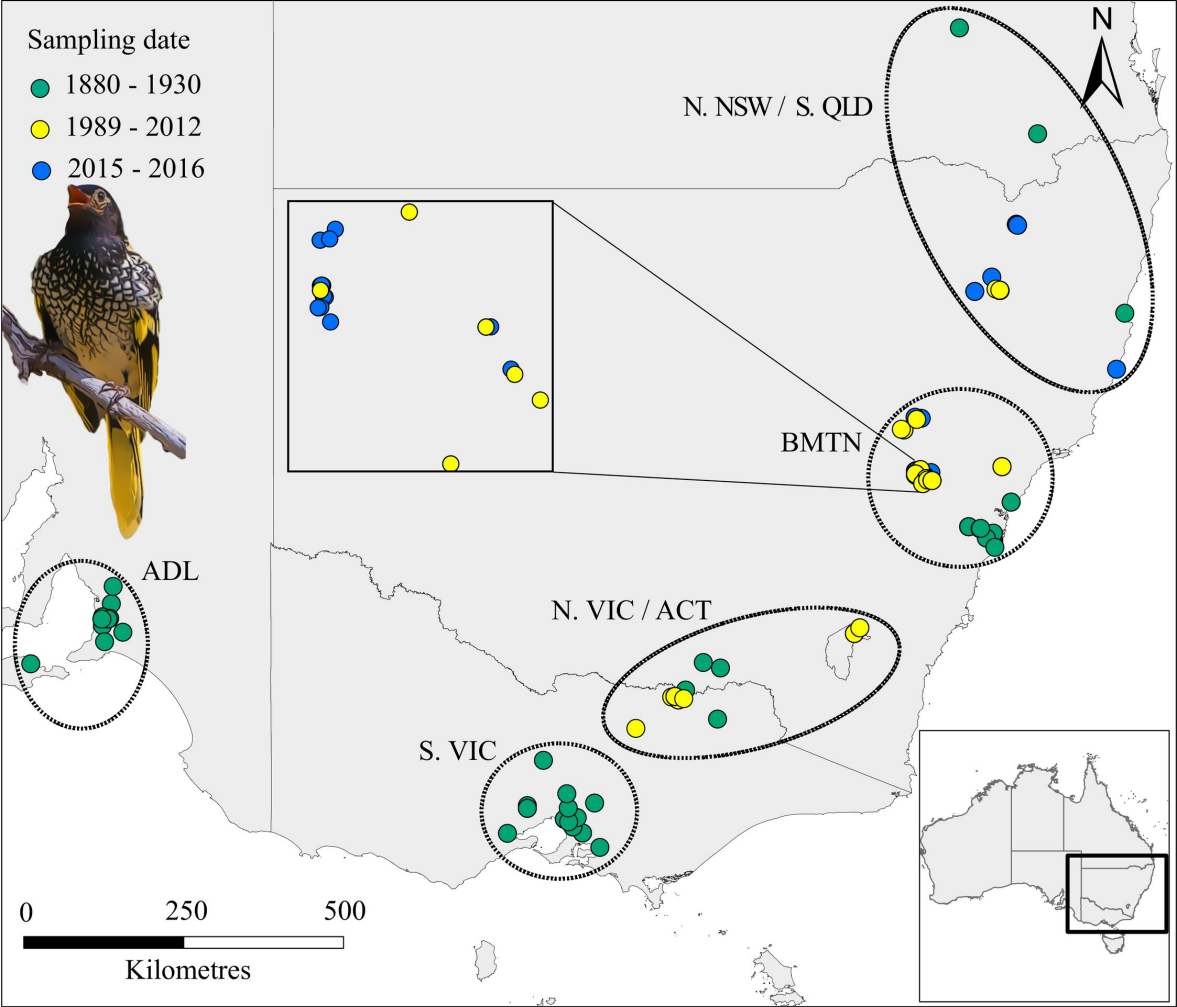
Location of regent honeyeater DNA samples by geographic region (denoted by ellipses) and sampling date (i.e. historic n = 62, recent n = 104, and current n = 52). Regional *a-priori* population abbreviations are: Adelaide (ADL), southern Victoria (S.VIC), northern Victoria / Australian Capital Territory (N.VIC), greater Blue Mountains (BMTN) and northern New South Wales / southern Queensland (N.NSW). Inset: location of recent and current samples within Capertee Valley, BMTN- the core breeding area for the remaining wild population [31]. Due to scale and spatial clustering of samples, not all individuals are visible on the map. See S1 Table for further information.

The regent honeyeater *Anthochaera phrygia* is a critically endangered Australian songbird with an average generation time of 5.8 yrs [25], for which contemporary population data is severely limited. Regent honeyeaters were widespread throughout their former range extending from Adelaide to southern Queensland [26] (Fig 1). Historical ecological data is lacking, but records document ‘immense flocks’ undertaking ‘semi-nomadic wanderings’ to track seasonal shifts in flowering *Eucalyptus* tree species [26]. Regent honeyeaters breed in lowland woodlands [26], which have been extensively cleared since the 1940s [27]. Concurrently, the regent honeyeater has undergone rapid population decline and range contraction [28]. Regent honeyeaters were extinct in South Australia by the 1980’s [29] (Fig 1). Since then, nest records have waned from breeding sites in Victoria, the Australian Capital Territory, and central-western New South Wales [30]. Although sightings of non-breeding birds are distributed throughout their 600,000 km^2^ range, current known breeding activity is almost exclusively restricted to two regions of NSW: the greater Blue Mountains (BMTN), and the Northern Tablelands (N. NSW, Fig 1) [31].

Using 10 microsatellite markers and 108 samples collected throughout the species’ recent range between 1989–2012 (Fig 1), Kvistad *et al*. [13] found no evidence that population decline and fragmentation had led to recent population structure, estimating the effective population size at 2012 to be 68–147 with the temporal method. Whilst these results likely reflect biological reality, it is possible that loss of genetic diversity and changes in population structure were not detected because they occurred before or after the sampling period, or through a lack of genetic resolution in microsatellite markers or sample size [13].

We build on the work of Kvistad *et al*. [13] to place the regent honeyeater’s contemporary population genomics in an historical context by: (1) incorporating 64 museum specimens sampled from throughout the species’ former range; (2) collecting 50 ‘current’ samples from the remaining wild population; and (3) using next generation sequencing to construct and analyse a SNP database of the species. Our sampling covers three distinct time periods: ‘historic’ (1879–1960), when the species was abundant and widespread (no robust population estimate), ‘recent’ (1989–2012) when the estimated population declined from *ca*. 2000 to 500, and ‘current’ (2015–2016) with an estimated 250–400 individuals largely restricted to two breeding regions separated by > 350 km (Fig 1) [31]. We aimed to quantify the impact of widespread habitat loss on the population genomic structure, diversity and effective population size of the wild regent honeyeater populations over these time periods.

## Materials and methods

### Sample collection and DNA extraction

We included a total of 230 samples in this study from 5 geographic regions (*a-priori* populations, Fig 1). The collection date of 74 museum specimens from which we obtained toe-pad samples ranged from 1879 to 1960 (S1 Table). We sourced recent samples from Kvistad *et al*. [13] in the form of extracted DNA from 104 wild birds captured between 1989–2012. We located current regent honeyeaters by developing a range-wide survey protocol [31]. With a single 9 m mist net, we captured 48 adults and one juvenile in 2015 and 2016. We also included a tissue sample from one dead nestling. Using standard brachial venepuncture, we sampled blood from all birds, apart for 10 adults from which we collected body feathers shed during the capture and handling process. We stored all current samples in 70% ethanol. Sample metadata are provided in S1 Table. Our contemporary samples were collected under Australian National University (ANU) animal ethics permit #A2015/28, Australian bird and bat banding service authorities #3192 and #2633, New South Wales scientific licences #SL101580 #SL100850 and Victorian scientific licence #10008288.

We extracted DNA at the EcoGenomics and Bioinformatics Laboratory at the ANU, where avian DNA extraction has not been implemented previously. For aDNA extraction from museum samples and for DNA extraction from contemporary feather samples, we used Qiagen DNeasy Blood and Tissue kits (QIAGEN, California), with modifications to the manufacturer’s instructions per Olah *et al*. [32]. DNA extraction from blood followed standard salting out protocol [33].

### Probe preparation using ddRAD

Laboratory procedures followed the hybridization restriction associated DNA (hyRAD) protocol of Suchan *et al*. [23] with some modifications (S1 Fig). Since a regent honeyeater whole genome was not available, we downloaded from GenBank all genetic sequences of *Acanthorhynchus tenuirostris*, the closest relative species, to calculate the average G:C content (0.392) for the simulation of a 1.2 gb genome with the ‘SimRAD’ package [34]. In order to reach the necessary amount of SNP loci, we used SimRAD, applying *in-silico* digestion on the simulated genome, and estimating the target size for 155–235 bp without adapter regions. For the *ddRAD library* preparation (S1 Fig), we selected five samples with high quality genomic DNA (1,500 ng each), representing the species recent/current distribution (N012/BMTN, N015/N.NSW, N039/N.NSW, R031/N.VIC, and R038/ACT, S1 Table), and digested individually with 15 U SphI (NEB) and 15 U MluCI (NEB). After confirming digestion profiles using the LabChip GXII (Caliper Life Sciences, USA), we purified DNA using Sera-Mag SpeedBeads (GE Healthcare) in bead:sample ratio of 2:1. Following ligation of short adapters containing only primer binding sites for amplification (see online protocol), we pooled the five samples and bead purified as above. We used a LabChip XT fractionation system (Caliper Life Sciences, USA) to size-select the ligation product using a DNA 300 Chip for isolation of fragments between 210−290 bp (including adapter regions). We PCR amplified the size-selected DNA in 20 replicates using MyTaq HS Mix (Bioline) for 38 cycles. We then pooled amplicons, purified them with a bead:sample ratio of 1:1, and quantitated with a Qubit 3.0 fluorometer (Life Technologies). In order to yield sufficient amplified product (500−1,000 ng/capture), we re-amplified some of the PCR products, achieving a total of 30,000 ng DNA after purification. We converted the whole ddRAD library into probes by removing the short adapter sequences using the same restriction enzymes, and purifying with a bead:sample ratio of 1.5 : 1. Finally, we biotinylated the DNA fragments with DecaLabel DNA Labeling Kit (Thermo Fisher Scientific), followed by an additional purification using bead:sample ratio of 1.5:1. We used these DNA probes as ‘baits’ in the subsequent hybridization sequence capture.

### Genomic library preparation

We prepared the whole genome library using all available samples, including those used for the ddRAD probes (S1 Fig). We first quantitated samples by fluorometry using the Quant-iT dsDNA Assay kit (Invitrogen). The 156 contemporary samples we then sheared using a Bioruptor sonicator (Diagenode) with a variable number of cycles depending on the sample age and level of degradation. We sheared samples to a mode of 300 bp and visualised by GXII chip electrophoresis. Where possible, we used the same quantity of DNA samples while preparing the whole genome library, which ranged between 5–750 ng/sample. With some modifications, we followed the methods of Suchan *et al*. [23] to prepare libraries.

We hybridized the DNA probes with the whole genome library template (S1 Fig). For hybridization, we pooled the 230 samples in 10 equimolar groups, each containing 23 samples of similar age. Each of the 10 hybridization mix contained 500–1,000 ng of each pooled DNA library group and 500–1,000 ng of the biotinylated probes. The hybridization buffer contained 1 mg/ml of Chicken Hybloc Cot-1 DNA (Applied Genetics Laboratories, USA), 2 μM of each blocking oligo, 6x SSC, 50 mM EDTA, 1% SDS, and 2 x Denhardt’s solution. We denatured each mix at 95°C for 5 min and incubated at 65°C for 48–72 hours. Using streptavidin Dynabeads M-280 (Life Technologies), we captured the probes that hybridized with the targeted fragments and removed target DNA from the probes and beads and purified with SSC/SDS buffer. We enriched the resultant library via 15 cycles of PCR amplification using NEBNext High-Fidelity PCR Master Mix (NEB), and performed a final purification with bead:sample ratio of 1:1 on each capture. With GXII assays, we verified the profile and concentration of each captured library, and pooled the 10 hybridization-enriched and reamplified pools together in equimolar ratios.

### Scaffold sequence

Using TruSeq Nano DNA LT Kit (Illumina), we made a 550 bp average insert library with a single contemporary sample of high molecular weight (N046/BMTN; NCBI BioSample number: SAMN12070213).). We spiked this sample into the hyRAD library at approximately 40% for the dual purpose of increasing the complexity of template sequence in the subsequent HiSeq lane and providing a low coverage whole genome scaffold sequence for a single individual. We sequenced the library using 100 bp paired-end option on a single lane of the Illumina HiSeq 2500 sequencing machine at the ANU Biomolecular Resources Facility.

### Bioinformatics pipeline

We processed the sequenced data using the Computational Biology and Bioinformatics Unit (CBBU) Linux-based computational cluster at the Research School of Biology, ANU. A total of 504,269,284 raw sequencing reads were received. We demultiplexed barcodes using the ‘fastq-multx’ function of the ‘ea-utils’ package [35], resulting in 399,241,340 unique sequences from 230 individuals, and 98,426,238 reads from the single sample whole genome. The average number of PE reads per individual were 1,158,622 bp for historic-, 1,848,232 bp for recent-, and 2,332,445 bp for current samples. We used FastQC [36] to check the quality of raw sequencing reads and Trimmomatic [37] to trim reads.

We constructed a whole genome scaffold from the single individual library using SOAPdenovo2 [38] with *kmer* size of 23. Following the HaploMerger2 pipeline [39] using default parameters, we polished the genome scaffold and closed gaps. We then blasted the final scaffold against the NCBI ‘nt’ database and the external contamination was negligible (1.4% of reads with at least one hit). We deposited the generated whole genome scaffold to NCBI (accession number: VJPA00000000) as part of the BioProject PRJNA549051.

For reference mapping of the hyRAD unique sequences, we used the assembled contigs and the BWA-MEM aligner [40]. We used the MarkDuplicates tool from the Picard toolkit (http://broadinstitute.github.io/picard), to remove PCR duplicates and mapDamage 2.0 [41] to rescale base quality scores of putatively post-mortem damaged bases in the museum samples. Finally, we used Freebayes [42] to produce an initial set of 3,667,699 raw SNP sites. The structure of the sequenced genomic library and the full laboratory protocol is available online at https://www.protocols.io/view/hyrad-for-birds-mt2c6qe.

### Sample and SNP filtering

We implemented initial SNP filtering using VCFtools [43], following criteria published in Suchan et al. [23]. We only retained variants that were biallelic, present in at least 50% of individuals, had a sequencing depth between 5 and 1,000, a minimum quality score of 30, and a minimum minor allele frequency of 5%. We also removed indels and 16 samples that contained more than 90% missing SNP data. Because the proportion of missing data was higher in the museum samples ($\bar{x}$ 26%) than the contemporary samples ($\bar{x}$ 7%) after initial filtering, we increased the minimum sequencing depth threshold to 10 for all spatial analyses. For analyses of temporal change in genetic diversity, we increased the minimum sequencing depth to 15 and capped the maximum sequencing depth at 100 (S2 and S3 Figs). We then used package *dartR* v1.0 [44] to remove remaining loci that were present in < 75% of samples to minimise the temporal bias in data quality. A comprehensive summary of all filtering steps and the final SNP datasets used for each analysis is provided in S1 File. Unless otherwise stated, we conducted all analyses in R v3.4.3 [45]. An annotated R-script is available in the supplementary material (S2 File). We deposited all SNP data to the open-access European Variation Archive (EVA) under project PRJEB33704 and analyses ERZ1030386.

### Population genetic structure

We grouped samples into the three time periods (historic, recent and current) and assigned them to one of five *a-priori* regional populations (Fig 1, S1 Table). We based assignments on known breeding movements of colour-marked individuals within regional populations and the rarity of known movements between these populations (S4 Fig). A lack of a current N.VIC population reflects a failure of monitoring to detect regent honeyeaters there [31]. Unless otherwise stated, we ran all spatial population genetic analyses separately for the contemporary (recent and current) and historic datasets.

We first explored population structure using *fastSTRUCTURE* [46] and discriminant analysis of principal components (DAPC) [47]. In order to choose the appropriate number of model components that explain structure in the dataset in *fastSTRUCTURE*, we first ran the algorithm with the number of genetic clusters (K) from 1–10. We used a built-in utility tool to parse through the outputs and determine the cluster size that best explained structure in the data. We visualised the expected admixture proportions inferred by *fastSTRUCTURE* using Distruct plots [48]. Given the sensitivity of this analysis to missing data [46], we only ran it on the contemporary dataset. We used *adegenet* v 2.1.1 [47] to implement the DAPC. We allowed the package first to infer the number of clusters in the data using the ‘find.clusters’ function, but for subsequent analysis we based the number of clusters on the number of *a-priori* populations (i.e. 5). We used the optimal *a*-score and cross-validation per Jombart [47] to determine the number of retained principal components (PCs) and tested the capacity of the DAPC to correctly assign individuals to *a-priori* populations with supplementary individuals (historic n = 15, contemporary n = 50) that were excluded from the model.

We estimated significance of genomic differentiation at the population level via 999 permutations and *P*-values corrected for false discovery rate (FDR). We calculated Weir and Cockerham pairwise *F*_ST_ estimates between *a-priori* populations using *stAMPP* v1.5.1 [49], with significance estimated via 10,000 permutations and FDR correction. To assess temporal change in genetic isolation by distance at the *a-priori* population level, we fitted linear models of all standardised pairwise *F*_*ST*_ values against the log-transformed mean distance between samples in each population [50]. We then constructed an unrooted, bootstrapped (n = 10,000) dendrogram based on Prevosti’s genetic distance (which can handle missing data) in *poppr* v2.8 [51], to determine whether *a-priori* populations clustered by geographic distance.

To assess temporal changes in genetic isolation at the individual level, we used correlograms of genetic distance calculated in GenAlEx v6.503 [52], with significance of genetic distance (r) estimated via 999 random permutations. Using *poppr*, we counted the number of private alleles within each *a-priori* historical population. We ran an analysis of molecular variance (AMOVA), also using *poppr*, to determine the proportion of total molecular variance explained by differentiation between the five *a-priori* populations (Fig 1), testing a null hypothesis of no genetic differentiation between them. Because AMOVA cannot handle missing date, using the ‘missingno’ function we replaced remaining missing data with mean allele values.

### Genetic diversity

Using *Hierfstat* v0.04–22 [53], we calculated observed (H_O_) and expected (H_E_) individual heterozygosity, rarefied allelic richness (A_R_), and population-level inbreeding (F_IS_) at each time period. We replaced missing data with mean allele values to offset the higher proportion of missing data in the historic sample but, to assess the robustness of the results, we also evaluated these metrics without replacing missing data (S1 File, S14 Fig). We compared levels of historic, recent and current observed heterozygosity between individuals using Mann-Whitney U-tests. We examined pairwise differences in H_O_, H_E_, and A_R_ between the three time periods using 10,000 permutations, correcting *P*-values for FDR. We then calculated individual-level inbreeding at each time period using the *g*_*2*_ method via package *inbreedR* v0.3.2 [54]. To assess the potential for sampling bias to be introduced by lower quality data derived from the museum samples, we evaluated the effect of read depth on genetic diversity measures (H_O_, H_E_ and minor allele frequency, MAF) by visualising the mean value of each measure at sequence depths from 10 to 100 for each time period (S5–S7 Figs).

We counted the number of private alleles within each time period using *poppr*.

### Effective population size

To estimate effective population sizes in the recent and current population we used *NeEstimato*r v2.1 [55] with the linkage disequilibrium (LD) method [56]. We assumed random mating and calculated 95% confidence intervals using ‘jackknifing’ among pairs of loci. For additional, coalescent-based demographic analysis we used *BEAST* v2.4.8 [57, 58]. We performed a total of 10 Markov chain Monte Carlo (MCMC) runs, each for 1 billion samples to ensure convergence of the chains, using known sampling dates and the Jukes-Cantor substitution model. After examining the likelihood traces of each chain in *Tracer* v1.6.0 [59], we chose appropriate burn-in percentages, ranging from 30–90%, to construct extended Bayesian skyline plots, first for all samples and then again with only the contemporary samples.

## Results

The size of the regent honeyeater whole genome scaffold was 815,195,808 base pairs in 265,122 contigs (N50 = 4,482 bp). Average base misincorporations due to postmortem nucleotide damage per museum sample reads was 549,844 base pairs, representing 1.8% of the average base pair reads per museum sample (S8 and S9 Figs). After aligning the hyRAD sequences to this genome scaffold and initial filtering, the dataset contained 3,524 biallelic SNPs. After implementing stricter filtering criteria on minimum sequence depth, the dataset contained 215 individuals (62 historic, 153 contemporary). For spatial analyses, the dataset contained 1,246 SNPs ($\bar{x}$ 5.9% missing contemporary data, $\bar{x}$ 24% missing historic data) The dataset for temporal trends in genetic diversity contained 555 SNPs and 212 individuals (151 contemporary, $\bar{x}$ 17% missing data and 51 museum, $\bar{x}$ 39% missing data) prior to replacement with mean allele values (S1 File).

### Population structure

**Fig 2:**
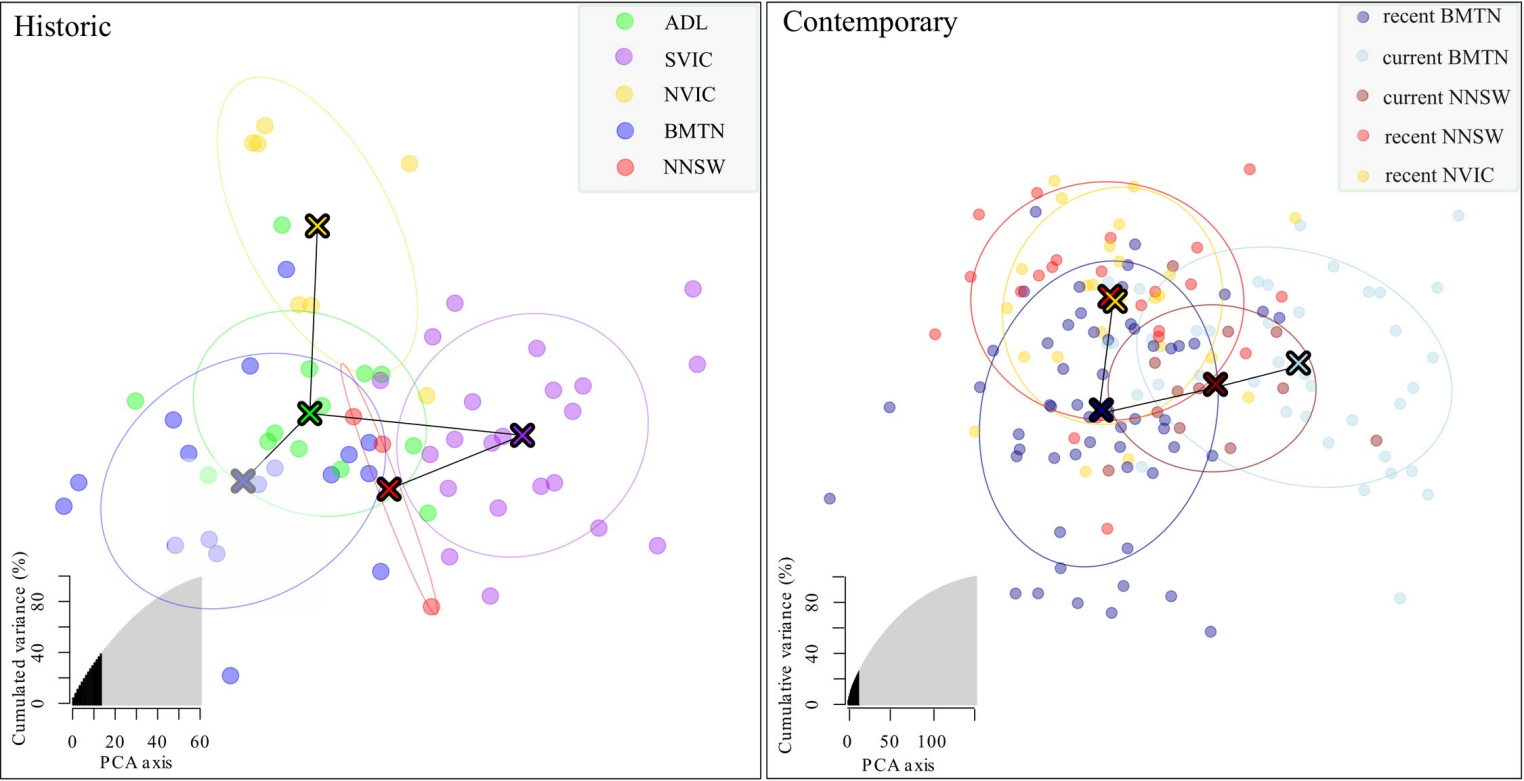
Discriminant analysis of principle component (DAPC) plots for historic and contemporary regent honeyeater samples by *a-priori* populations. Inset figures show proportion of total variance explained with the number of retained principle components. See S10 Fig for associated assignment plots.

When the contemporary samples were analysed in *fastSTRUCTURE* (i.e. three recent and two current *a-priori* populations), model complexity that maximised marginal likelihood was one (K = 1). The number of components used to explain structure in the data was four (K = 4), indicating that the true number of K was between these values. When the results were plotted, only K = 3 and 4 showed detectable structure, but this was explained by neither population nor time period. DAPC found weak clustering of individuals by populations in both the historic and contemporary samples (Fig 2 and S10 Fig). Discriminant analysis with all PCs initially retained also supported the existence of one cluster within the contemporary data (delta Bayesian Information Criterion, ΔBIC, was 1.29 for 1–2 clusters and 8.03 for 1–5 clusters). The number of retained PCs that maximised the *ɑ*-score was 11–14 and the number that minimised root mean squared error (RMSe) via cross-validation was 14. Of 50 supplementary individuals, only 26% were correctly assigned by the model to their *a-priori* populations (Fig 2 and S10 Fig). DAPC also supported the existence of just one cluster in the historic data (ΔBIC was 1.59 for 1–2 clusters, and 8.87 for 1–5 clusters). The number of retained PCs that maximised the *ɑ*-score and minimised RMSe was 9 and 10, respectively. Of 15 supplementary individuals, 27% were correctly assigned to their historic *a-priori* population.

**Fig 3:**
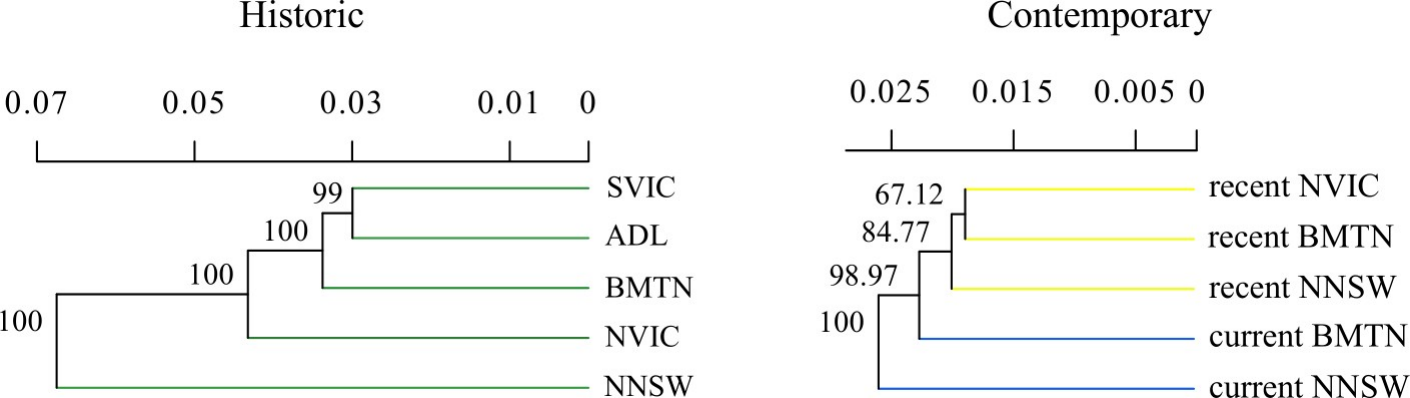
Bootstrapped dendrogram of historic and contemporary samples by *a-priori* regent honeyeater populations, based on Prevosti’s genetic distance. Note divergence scales differ between trees.

**Fig 4:**
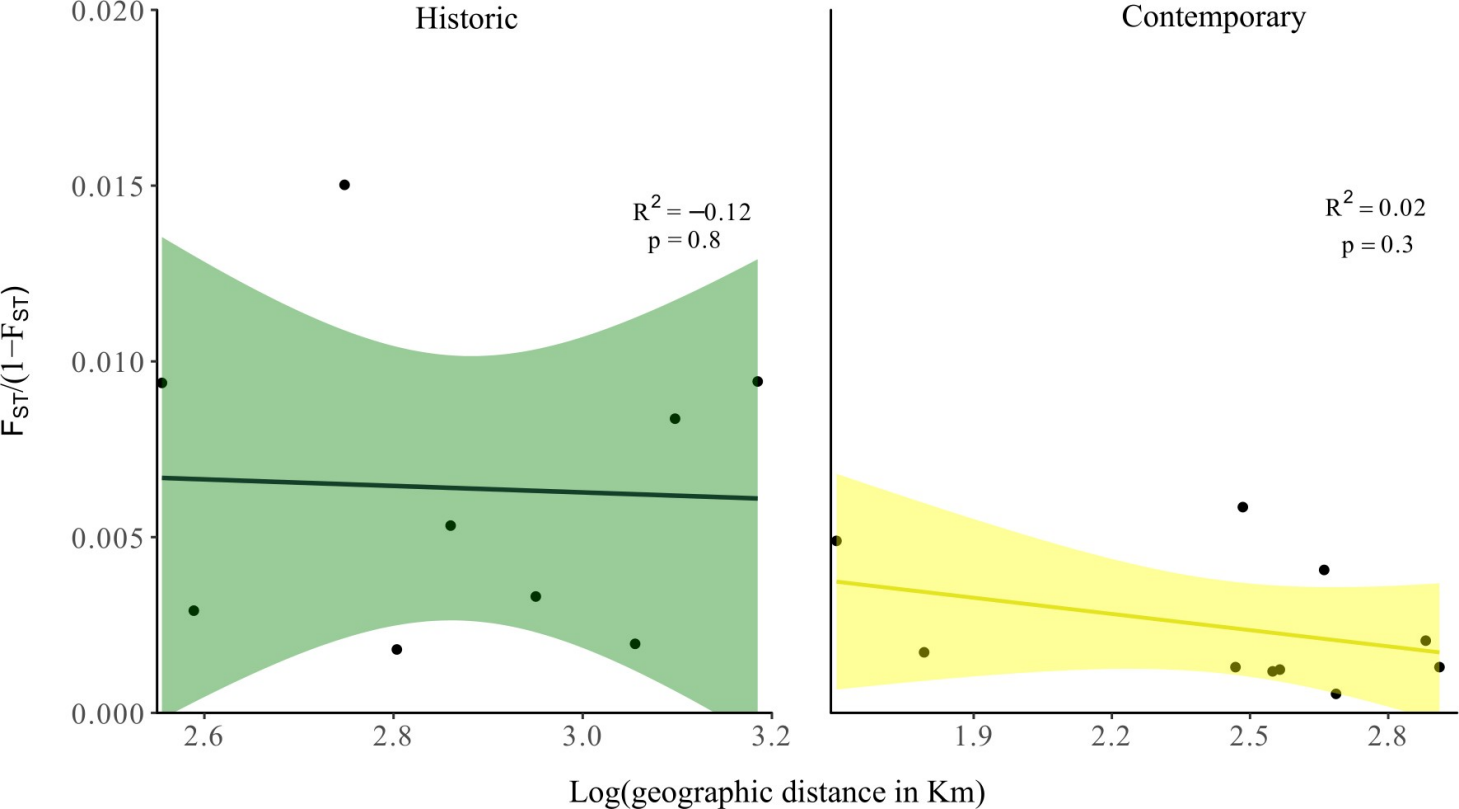
Standardized effect of geographic distance on *a-priori* population-level genetic differentiation in historic and contemporary (recent and current) regent honeyeater samples.

**Fig 5:**
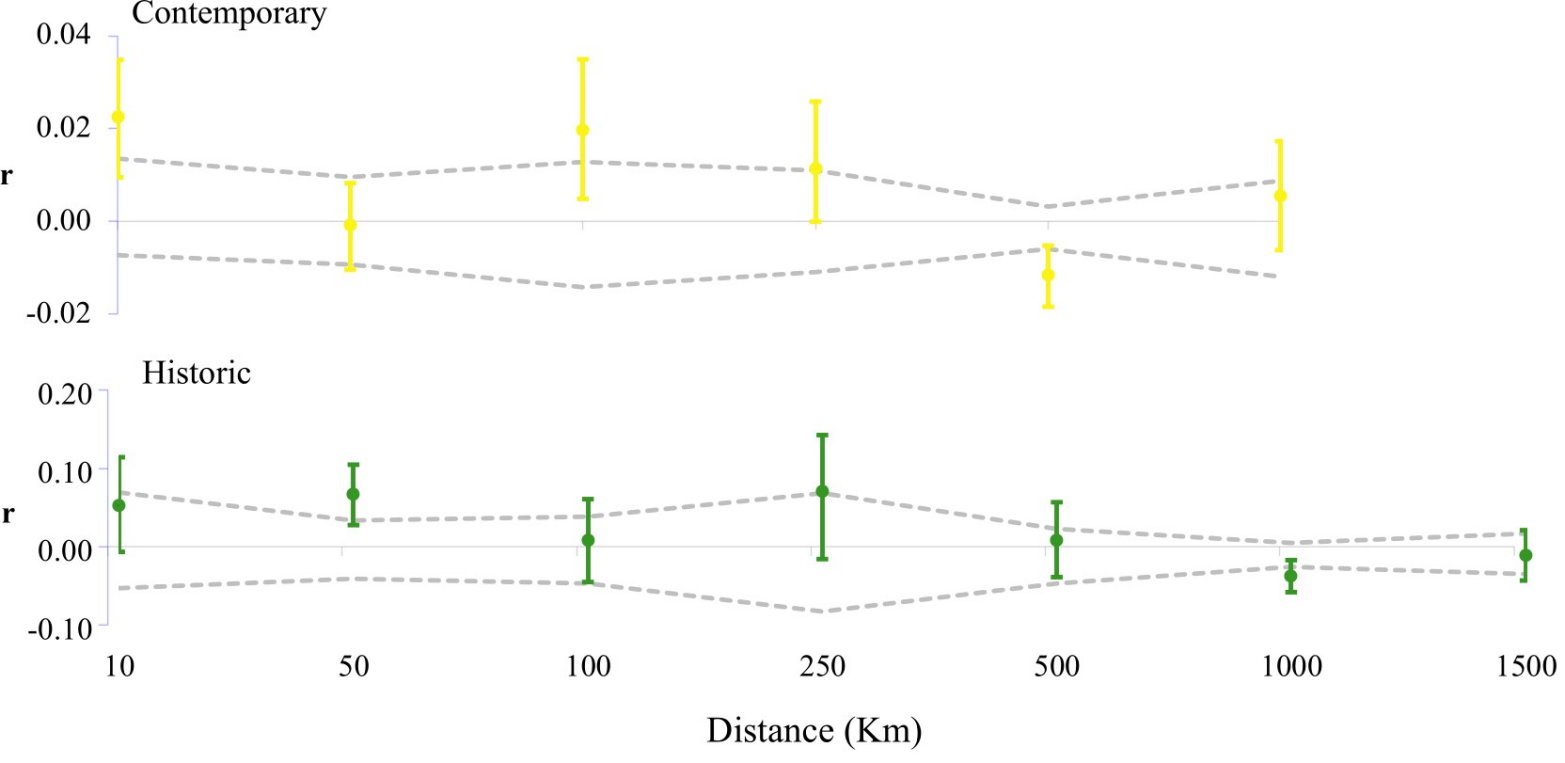
Spatial autocorrelation (r) in genomic similarity at varying distance classes within the historic and contemporary regent honeyeater population. Error bars (95% confidence intervals) at each distance class estimated via 999 bootstraps of sample pairs. Dashed lines denote simulated 95% upper and lower confidence intervals, assuming random distribution of regent honeyeaters within each time period.

Dendrograms revealed both historic and contemporary *a-priori* populations largely clustered by geographic distance with high bootstrap support (Fig 3). Linear models of standardized *F*_ST_ and correlograms of relatedness suggested genomic divergence estimates between both *a-priori* populations and individuals changed little with increasing geographic distance (Figs 1, 4 and 5).

AMOVA revealed weak but significant genomic differentiation between both historic and contemporary *a-priori* populations (Historic Sigma = 0.64, std.Obs = 3.93, *P*_*adj*_ = 0.012; Contemporary Sigma = 0.82, std,Obs = 6.69, *P*_*adj*_ = 0.003, S11 Fig), but the proportion of total molecular variance attributable to variation amongst populations was very small (Historic = 0.53%, Contemporary = 0.31%). Pairwise *F*_ST_ estimates were also very small between all historical *a-priori* populations. There were significant pairwise differences between southern Victoria and the Blue Mountains and between northern Victoria and both Blue Mountains and northern New South Wales (Table 1A). There were weak but significant genomic differentiations between three contemporary *a-priori* populations, with the current Blue Mountains population appearing most distinct (Table 1B).

**Table 1:** Pairwise population genetic differentiation between historic (A) and contemporary (B) *a-priori* regent honeyeater populations (see Fig 1 for location information). *F*_ST_ values are below diagonal and FDR corrected *P* values are above diagonal. Sample sizes for each population are shown in parentheses. *Significant (*P* < 0.05) population genetic differentiation.

**A**	ADL	S.VIC	N.VIC	BMTN	N.NSW
ADL (13)		0.56	0.22	0.22	0.17
S.VIC (22)	-0.0001		0.19	0.003*	0.17
N.VIC (7)	0.0033	0.0029		0.03*	0.03*
BMTN (16)	0.0020	0.0053	0.0093		0.052
N.NSW (3)	0.0093	0.0083	0.024	0.0148	
**B**	Recent BMTN	Recent N.NSW	Recent N.VIC	Current BMTN	Current N.NSW
Recent BMTN (56)		0.052	0.154	0.00*	0.151
Recent N.NSW (22)	0.0013		0.99	0.00*	0.154
Recent N.VIC (23)	0.0005	-0.0021		0.00*	0.154
Current BMTN (37)	0.00487	0.0058	0.0041		0.154
Current N.NSW (12)	0.00123	0.0017	0.0013	0.0012	

Within the historic sample, private alleles were detected in Adelaide (ADL, 9 loci), northern Victoria (N.VIC, 2 loci), southern Victoria (S.VIC, 45 loci), northern New South Wales (N.NW, 10 loci) and the Blue Mountains (BMTN, 7 loci). Within the five contemporary populations, there was a single private allele present in two individuals in the current BMTN population. The number of private alleles within the contemporary population varied little with degree of filtering (S2 Table). Within the historic population, the number of private alleles varied with filtering extent, but the *proportion* of private alleles within each population remained consistent across filtering criteria (S2 Table). There were no private alleles when comparing across all three time periods (S2 Table).

### Genetic diversity

**Fig 6:**
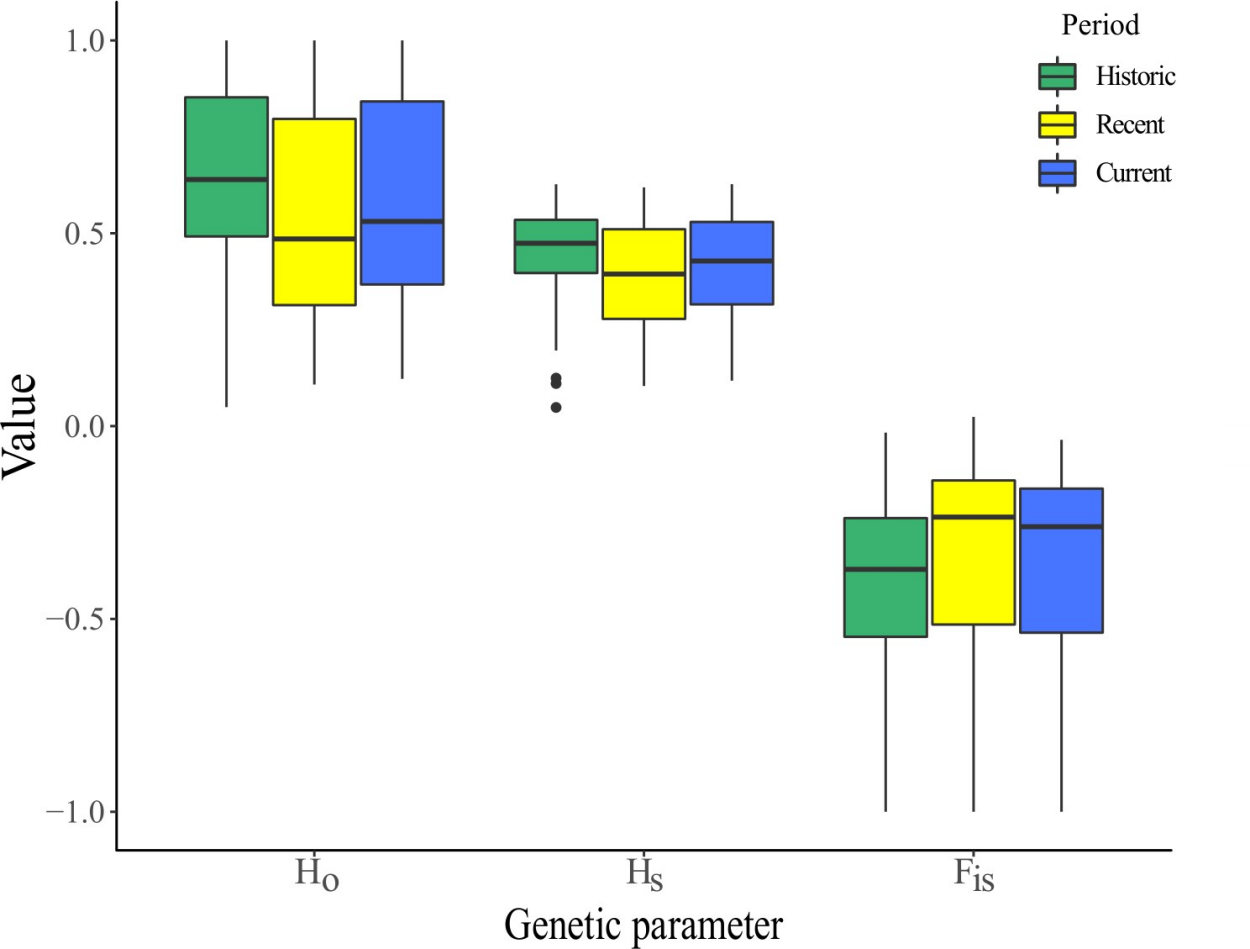
Temporal variation in heterozygosity (Ho & H_E_) and inbreeding (*F*_IS_) in the regent honeyeater population.

Observed and expected heterozygosities were higher in the historic population than in the recent (Mann-Whitney U test: W_HO_ = 207500, *P*_HO_ = < 0.001; W_HE_ = 195280, *P*_HE_ < 0.001) and current populations, (W_HO_ = 199920, *P*_HO_ = < 0.001; W_HE_ = 183810, *P*_HE_ < 0.001). Heterozygosity measures differed slightly between the recent and current populations (W_HO_ = 144680, *P*_HO_ = 0.08; W_HE_ = 142110, *P*_HE_ = 0.03). *F*_IS_ was lower in the historic population than in the recent (W_FIS_ = 39742, *P*_FIS_ < .001) and current populations (W_FIS_ = 97005, *P*_FIS_ < .001), but there was no difference in inbreeding between the contemporary (i.e. recent and current) populations (W_FIS_ = 160230, *P*_FIS_ = 0.24). Allelic richness in the historic population was higher than in the recent (W_AR_ = 298460, *P*_AR_ = < 0.001) and current populations (W_AR_ = 281320, *P*_HO_ = < 0.001)Frequency distributions of estimated individual-level inbreeding coefficients (F) were low, with median *g*_*2*_ values ranging from 0.003 (current) to 0.008 (historic). Confidence intervals (95%) generated via bootstrapping broadly overlapped across the three time periods (S12 Fig). There was no evidence of differences or consistent declines in genetic diversity metrics within the contemporary populations (S13 Fig). Overall, spatio-temporal differences in all genetic diversity metrics were very small (Fig 6, S12 and S13 Figs). Estimates of temporal changes in the genetic diversity metrics were also robust to variation in filtering criteria and presence / absence of computed missing data (S1 File, S14 Fig).

### Effective population size

All model runs in *NeEstimator* resulted in infinite population size estimates. Similarly, convergence was difficult to achieve in some chains in *BEAST*. At all ranges of burn-in, skyline plots depicted similar trends in population size, suggesting an increase in population size over the past 500 years (S15 Fig). Although confidence intervals showed some evidence of a decrease in population size commencing around 35 years ago, a decrease in effective population size was not detectable.

## Discussion

Using a large, spatially-stratified dataset spanning over 130 years, we conducted a comprehensive population genomic analysis of the impact of severe population decline and range contraction in the regent honeyeater. We obtained thousands of SNPs from museum samples from as early as 1879. Despite evidence of very weak population genetic differentiation throughout their vast former range, the regent honeyeater population appears to have comprised a single, intermixing population prior to their rapid decline. Thus, we provide genomic evidence that regent honeyeaters have historically been a highly mobile species and that long-distance nomadic movements within the contemporary population are unlikely to be an obligate response to severe habitat loss. We confirm that the remaining wild population still represents a single management unit [19], despite severe population decline and range fragmentation. Population decline has led to minimal detectable loss of genetic diversity in the remaining population.

### Spatial genomic structure over time

Despite evidence that the regent honeyeater population did, and still does, represent a single genetic management unit, this does not imply that the population was, and still is panmictic [60]. Complementary spatial analyses indicated a degree of weak or very weak genetic differentiation within the historic and contemporary populations, respectively. Given a minimum distance of 2,000 km between the species’ historic north-south range boundary, and an historic range of over 600,000 km^2^, a degree of isolation-by-distance is perhaps unsurprising. Within the current samples, there was little genomic differentiation between the Blue Mountains and northern New South Wales populations, despite these small breeding populations (potentially < 50 remaining individuals in N.NSW) being separated by over 300 km for over two decades (Birdlife Australia, unpublished data) and substantial song differences between them [61]. Given the rapid rate of population decline, our genetic data lend support to a scenario whereby local extinction and associated breeding range fragmentation may be outpacing the establishment of spatial genomic differentiation in the remaining regent honeyeater population.

### Spatio-temporal patterns of genetic diversity

We found evidence that population decline has led to loss of heterozygosity and allelic richness within the population, but the magnitude of this loss was very small, relative to the magnitude of population decline. Statistical tests for temporal changes in genomic diversity were very sensitive given the large sample sizes and visualisation of the data showed these temporal differences were indeed very small (Fig 6). Due to the poorer quality of data derived from the historic samples (higher proportion of missing data, lower mean sequence depth and more random degradation of DNA fragments), and despite attempts to minimise these differences through stricter filtering criteria (S1 File, S2 and S3 Figs) and interpolation of missing data, our efforts to quantify the overall magnitude of genetic diversity loss in the regent honeyeater should be interpreted with caution. Rather than reflecting a true biological pattern, apparent homozygote excess in some historic samples could also be due to allelic dropout, whereby one allele fails to amplify in degraded heterozygote samples, resulting in a bias in favour of homozygotes [62, 63].

### Changes in effective population size

Despite a rapid and severe population decline, we were unable to derive reliable estimates of historic and current effective population sizes. Given their generation length of 5.8 years, regent honeyeater population decline may have occurred too rapidly to provide a detectable genomic signal at present [64]. Kivstad *et al*. [13] estimated the contemporary effective population size to be 149 (95% parametric CI = 68–147) with a microsatellite dataset. Since simulations show that *NeEstimator* can derive robust population estimates using just 200 SNPs [54], it should have been possible to derive effective population size estimates with our dataset. Sampling error (e.g. sampling related individuals or multiple individuals from the same population) or biases associated with large SNP datasets could have led to an infinite population estimate [54] and coalescent-based population estimates derived from *BEAST* support this supposition. There was more missing data in the historic sample, which could explain an apparent increase in population size over the past 500 years depicted by the skyline plot. Although skyline plots can be confounded by the presence of population structure [65], our spatial analyses suggest that this should not have biased regent honeyeater population estimates.

### Implications for regent honeyeater conservation

Given the current wild regent honeyeater population represents a single genetic management unit, capacity for active genetic management of the remaining population through translocation or supplementation appears limited [21]. High mobility of regent honeyeaters means that artificial gene flow that could be initiated via translocation is not likely to benefit the species, because natural gene flow is already maximising adaptive capacity. Yet, similar to other nomadic resource specialists like the swift parrot *Lathamus discolor* [7], we found no genomic evidence of spatial population structure in regent honeyeaters to buffer the species against threats that contribute to ongoing population decline [66]. Preservation of adaptive potential within the remaining population would appear to be best achieved through indirect conservation actions, including urgent attempts to increase breeding success, breeding participation, halting the loss of existing breeding habitat, and large-scale restoration of lost breeding habitat [31, 66].

### Implications for spatial population genetic studies using hyRAD

Genomic analysis of museum specimens is a rapidly expanding area of research [12, 23]. We therefore aimed to determine what spatio-temporal population genomic questions could be answered with a relatively large, long-term SNP dataset. With 230 individuals stratified in space and time, we assumed the sample should have been sufficient to answer the questions we were unable to answer, had data quality permitted [67, 68].

Due to a higher proportion of missing data in museum samples despite rigorous filtering [69], the historic and contemporary datasets were not of equal quality (S2 and S3 Figs). Temporal sampling of individuals was non-random throughout the range due to ongoing population decline, meaning only museum samples were available from Adelaide and southern Victoria. We were only able to generate probes from good quality DNA samples of the birds using ddRAD techniques, and given the different distribution of extinct and contemporary populations, this might have introduced some unavoidable ascertainment bias. Separating samples by time (museum vs. contemporary) did not hinder our capacity to test for spatial population structure however. With nearly 600 SNPs post-filtering, we were able to derive reliable spatial inferences from the museum data. However, potential allelic dropout and non-random fragmentation of aDNA likely hindered the capacity of the dataset to show evidence of temporal changes in genetic diversity and population size [62, 64]. Future studies with hyRAD techniques might benefit from a preliminary whole genome sequencing of the study species if not available (e.g. even with Nanopore MinION technology), which would help not only with aligning the short reads but also to better target the size range of the probes for more balanced SNPs / sequencing depths in the final dataset. Quantifying genetic diversity loss using museum specimens, though highly desirable in terms of deriving more accurate estimates of genetic threats in endangered species [14], appears to be an ongoing challenge [24]. We encourage close scrutiny of hyRAD datasets for spatio-temporal biases in data quality, rigorous filtering appropriate for the questions being asked, and testing the robustness of results to variation in filtering extent.

## Supporting information

S1 Table Regent honeyeater sample metadata. (DOCX)

S2 Table Summary of private allele counts in the regent honeyeater hyRAD dataset over time, within *a-priori* population and under various filtering criteria. (DOCX)

S1 Fig Structure of the genomic library preparations. (A) ddRAD libraries and (B) whole genome libraries, hybridization, and sequencing.
(PDF)

S2 Fig Percentage of missing data in the regent honeyeater hyRAD dataset by sample and sampling period at minimum read depths of 5, 10, 15 and 20. (TIF)

S3 Fig Boxplots of sequence depth by sample and sampling period in the regent honeyeater hyRAD dataset at minimum read depths of (A) 10 and (B) 15.
(TIF)

S4 Fig Frequency distribution of straight-line distance between capture location and subsequent public resighting location of colour-marked regent honeyeaters between 1980 and 2016. Data collected from BirdLife Australia citizen science project (D.Ingwersen, unpublished data). Distances calculated using the measure tool in GoogleEarth.
(TIF)

S5 Fig Plots of genetic diversity measures (observed heterozygosity Ho, expected heterozygosity HE, and minor allele frequency, MAF) by minimum sequence depth threshold and sampling period in the regent honeyeater hyRAD dataset. (TIF)

S6 Fig Plot of moving average (mean) heterozygosity (± SE) by sampling period in the regent honeyeater hyRAD dataset at read depths from 10 to 40. Highlighted area shows significantly lower heterozygosity in the historic sample at read depths < 13, hence the use of min DP = 15 for temporal genetic diversity analyses.
(TIF)

S7 Fig Plot of observed v expected heterozygosity in the regent honeyeater hyRAD dataset. (A): Minimum sequence depth = 10, missingness. cutoff = 0.75. (B): Minimum sequence depth 15, maximum sequence depth 100, missingness. cutoff = 0.65.
(TIF)

S8 Fig Selection of assembled deamination plots for regent honeyeater hyRAD museum samples. (TIFF)

S9 Fig Frequency distribution of base misincorporation plots for regent honeyeater hyRAD museum samples. (TIF)

S10 Fig Discriminant Analysis of Principal Component (DAPC) assignment plots showing individual assignment probability to *a-priori* regent honeyeater populations for historic and contemporary samples. (TIF)

S11 Fig Analysis of molecular variance plots of observed (black points) and simulated (grey histograms) variation within and between samples and between historic (A) and contemporary (recent and current, B) *a-priori* regent honeyeater populations, with FDR correction.
(PDF)

S12 Fig Frequency distribution of estimated individual-level inbreeding (F), using the *g_2_* method, in individual regent honeyeaters by sampling period. Lateral error bars represent 95% confidence intervals estimated via 100 bootstraps of each sample.
(TIF)

S13 Fig Spatio-temporal variation in heterozygosity (HO and HE) and inbreeding (*F*IS, top) and allelic richness (AR, bottom) within contemporary *a-priori* regent honeyeater populations. Note no current data available for N.VIC.
(TIF)

S14 Fig Temporal variation in genetic diversity metrics heterozygosity (HO and HE) and inbreeding (*F*IS) within the regent honeyeater sample under different filtering scenarios of minimum read depth (range 5–15) and missing data replaced / not replaced with mean allele values. (TIFF)

S15 Fig Bayesian skyline plot of estimated regent honeyeater effective population size. (TIF)

S1 File Overview of filtering processes undertaken on the regent honeyeater hyRAD dataset, temporal differences in % missingness of each dataset and list of analyses performed on each dataset. (XLSX)

S2 File Annotated script for all regent honeyeater hyRAD data processing and analysis undertaken in R. See Methods and S1 File for further information.
(ZIP)
