# Supplementary material for: Genomic impact of severe population decline in a nomadic songbird
Source: PLoS One. 2019 Oct 24;14(10):e0223953. doi: 10.1371/journal.pone.0223953 (PMC6812763; doi:10.1371/journal.pone.0223953)
Supplement: S1 Table — (DOCX) [file pone.0223953.s001.docx]

| **Table S1.** Regent honeyeater sample metadata. | | | | | | |  |  |  |
| --- | --- | --- | --- | --- | --- | --- | --- | --- | --- |
|  |  |  |  |  |  |  |  |  |  |
| **SAMPLE ID** | **YEAR** | **POP** | **AGE** | **LOCATION** | **LOCATION NAME** | **SEX** | **SAMPLE TYPE** | **ABBBS BAND No.** | **COLLECTION** |
| N001 | 2015 | BMTN | current | Available upon request | Capertee | M | BLOOD | 053-11089 | Australian National University |
| N002 | 2015 | BMTN | current | Available upon request | Capertee | M | BLOOD | 053-12303 | Australian National University |
| N003 | 2015 | NNSW | current | Available upon request | Gwydir River | M | BLOOD | 053-11086 | Australian National University |
| N004 | 2015 | BMTN | current | Available upon request | Capertee | F | BLOOD | 053-12301 | Australian National University |
| N005 | 2015 | BMTN | current | Available upon request | Capertee | M | BLOOD | 053-12302 | Australian National University |
| N006 | 2015 | BMTN | current | Available upon request | Capertee | M | BLOOD | 053-11094 | Australian National University |
| N007 | 2015 | BMTN | current | Available upon request | Capertee | F | BLOOD | 053-11096 | Australian National University |
| N008 | 2015 | BMTN | current | Available upon request | Capertee | M | BLOOD | 053-11098 | Australian National University |
| N010 | 2015 | BMTN | current | Available upon request | Capertee | M | BLOOD | 053-11088 | Australian National University |
| N011 | 2015 | BMTN | current | Available upon request | Capertee | M | BLOOD | 053-11097 | Australian National University |
| N012* | 2015 | BMTN | current | Available upon request | Capertee | F | BLOOD | 053-12315 | Australian National University |
| N013 | 2015 | BMTN | current | Available upon request | Capertee | M | BLOOD | 053-12307 | Australian National University |
| N014 | 2015 | BMTN | current | Available upon request | Capertee | M | BLOOD | 053-11095 | Australian National University |
| N015* | 2015 | NNSW | current | Available upon request | Lake Cathie | M | BLOOD | 053-11087 | Australian National University |
| N016 | 2015 | BMTN | current | Available upon request | Capertee | M | BLOOD | 053-11093 | Australian National University |
| N017 | 2015 | BMTN | current | Available upon request | Capertee | M | BLOOD | 053-11091 | Australian National University |
| N018 | 2015 | BMTN | current | Available upon request | Capertee | F | BLOOD | 053-11090 | Australian National University |
| N019 | 2015 | BMTN | current | Available upon request | Capertee | M | BLOOD | 053-12312 | Australian National University |
| N020 | 2015 | BMTN | current | Available upon request | Capertee | F | BLOOD | 053-12310 | Australian National University |
| N021 | 2015 | BMTN | current | Available upon request | Capertee | M | BLOOD | 053-12313 | Australian National University |
| N022 | 2015 | BMTN | current | Available upon request | Capertee | M | BLOOD | 053-12308 | Australian National University |
| N023 | 2015 | BMTN | current | Available upon request | Capertee | F | BLOOD | 053-12311 | Australian National University |
| N024 | 2015 | BMTN | current | Available upon request | Capertee | F | BLOOD | 053-12314 | Australian National University |
| N025 | 2015 | BMTN | current | Available upon request | Capertee | M | BLOOD | 053-12309 | Australian National University |
| N026 | 2015 | BMTN | current | Available upon request | Capertee | F | BLOOD | 053-12304 | Australian National University |
| N027 | 2015 | BMTN | current | Available upon request | Capertee | M | BLOOD | 053-12306 | Australian National University |
| N028 | 2015 | BMTN | current | Available upon request | Capertee | M | BLOOD | 053-12305 | Australian National University |
| N029 | 2015 | BMTN | current | Available upon request | Capertee | M | FEATHER | 053-11100 | Australian National University |
| N030 | 2016 | BMTN | current | Available upon request | Capertee | F | BLOOD | 053-12316 | Australian National University |
| N031 | 2016 | BMTN | current | Available upon request | Capertee | F | FEATHER | 053-12317 | Australian National University |
| N033 | 2016 | BMTN | current | Available upon request | Capertee | M | BLOOD | 053-12319 | Australian National University |
| N034 | 2016 | BMTN | current | Available upon request | Capertee | M | BLOOD | 053-12320 | Australian National University |
| N035 | 2016 | BMTN | current | Available upon request | Capertee | M | BLOOD | 053-12321 | Australian National University |
| N036 | 2016 | BMTN | current | Available upon request | Capertee | M | BLOOD | 053-12322 | Australian National University |
| N037 | 2016 | BMTN | current | Available upon request | Capertee | F | FEATHER | 053-12323 | Australian National University |
| N038 | 2016 | NNSW | current | Available upon request | Severn River | F | FEATHER | 053-12324 | Australian National University |
| N039* | 2016 | NNSW | current | Available upon request | Severn River | M | BLOOD | 053-12325 | Australian National University |
| N040 | 2016 | NNSW | current | Available upon request | Severn River | F | BLOOD | 053-12326 | Australian National University |
| N041 | 2016 | NNSW | current | Available upon request | Severn River | M | FEATHER | 053-12327 | Australian National University |
| N042 | 2016 | NNSW | current | Available upon request | Severn River | M | FEATHER | 053-12328 | Australian National University |
| N043 | 2016 | BMTN | current | Available upon request | Capertee | F | BLOOD | 053-12329 | Australian National University |
| N044 | 2016 | NNSW | current | Available upon request | Severn River | M | BLOOD | 053-12330 | Australian National University |
| N045 | 2016 | NNSW | current | Available upon request | Severn River | M | FEATHER | 053-12331 | Australian National University |
| N046 | 2016 | BMTN | current | Available upon request | Capertee | F | BLOOD | 053-12332 | Australian National University |
| N047 | 2016 | BMTN | current | Available upon request | Capertee | M | BLOOD | 053-12333 | Australian National University |
| N048 | 2016 | NNSW | current | Available upon request | Barraba | M | BLOOD | 053-12334 | Australian National University |
| N049 | 2016 | NNSW | current | Available upon request | Barraba | M | FEATHER | 053-12335 | Australian National University |
| N050 | 2016 | NNSW | current | Available upon request | Severn River | U | TOE PAD | N/A | Australian National University |
| N051 | 2016 | BMTN | current | Available upon request | Goulburn River | M | FEATHER | 053-12336 | Australian National University |
| N052 | 2016 | BMTN | current | Available upon request | Goulburn River | M | FEATHER | 053-12337 | Australian National University |
| M001 | 1880 | ACT | historic | -N/A | N/A | M | TOE PAD | N/A | American Museum of Natural History |
| M002 | N/A | ADL | historic | -35.245,138.885 | Strathbalbyn | U | TOE PAD | N/A | American Museum of Natural History |
| M003 | 1900 | ADL | historic | -34.845,138.725 | Tree Gully | M | TOE PAD | N/A | American Museum of Natural History |
| M004 | 1904 | NVIC | historic | -36.061, 146.8 | N/A | F | TOE PAD | N/A | American Museum of Natural History |
| M006 | 1885 | BMTN | historic | -33.415, 151.379 | Gosford | F | TOE PAD | N/A | American Museum of Natural History |
| M007 | 1897 | BMTN | historic | -33.415, 151.379 | Gosford | F | TOE PAD | N/A | American Museum of Natural History |
| M008 | 1879 | BMTN | historic | -33.935, 151.129 | Cooks River | F | TOE PAD | N/A | American Museum of Natural History |
| M009 | 1879 | BMTN | historic | -33.935, 151.129 | Cooks River | F | TOE PAD | N/A | American Museum of Natural History |
| M010 | 1898 | BMTN | historic | -33.935, 151.129 | Cooks River | F | TOE PAD | N/A | American Museum of Natural History |
| M011 | 1893 | NVIC | historic | -35.671, 147.05 | N/A | M | TOE PAD | N/A | American Museum of Natural History |
| M012 | 1916 | BMTN | historic | -33.98, 151.1208 | Hogarah, Sydney | M | TOE PAD | N/A | American Museum of Natural History |
| M013 | 1916 | SVIC | historic | -38.071, 145.35 | N/A | U | TOE PAD | N/A | American Museum of Natural History |
| M014 | 1916 | SVIC | historic | -38.071, 145.35 | Bimberry, Manilara | U | TOE PAD | N/A | American Museum of Natural History |
| M015 | NA | BMTN | historic | -33.760, 150.780 | St. Marys | M | TOE PAD | N/A | American Museum of Natural History |
| M016 | NA | BMTN | historic | -33.760, 150.781 | St. Marys | M | TOE PAD | N/A | American Museum of Natural History |
| M017 | NA | BMTN | historic | -33.760, 150.782 | St. Marys | M | TOE PAD | N/A | American Museum of Natural History |
| M018 | NA | BMTN | historic | -33.760, 150.783 | St. Marys | F | TOE PAD | N/A | American Museum of Natural History |
| M019 | NA | BMTN | historic | -33.760, 150.783 | St. Marys | F | TOE PAD | N/A | American Museum of Natural History |
| M020 | NA | NNSW | historic | -30.755631,152.9759 | N/A | F | TOE PAD | N/A | American Museum of Natural History |
| M021 | NA | ACT | historic | N/A | N/A | U | TOE PAD | N/A | American Museum of Natural History |
| M022 | 1909 | ADL | historic | -35.0290, 138.6 | Blackwood | M | TOE PAD | N/A | American Museum of Natural History |
| M024 | 1901 | SVIC | historic | -37.986, 145.215 | Dandenong | M | TOE PAD | N/A | American Museum of Natural History |
| M025 | 1901 | SVIC | historic | -37.986, 145.215 | Dandenong | F | TOE PAD | N/A | American Museum of Natural History |
| M026 | 1908 | SVIC | historic | -37.856, 145.275 | Bayswater | F | TOE PAD | N/A | American Museum of Natural History |
| M028 | 1905 | SVIC | historic | -37.6866, 144.575 | Melton | M | TOE PAD | N/A | American Museum of Natural History |
| M029 | 1897 | SVIC | historic | -37.726, 144.575 | Castlemaine | U | TOE PAD | N/A | American Museum of Natural History |
| M030 | 1897 | SVIC | historic | -37.726, 144.575 | Castlemaine | U | TOE PAD | N/A | American Museum of Natural History |
| M031 | 1897 | SVIC | historic | -37.726, 144.575 | Castlemaine | U | TOE PAD | N/A | American Museum of Natural History |
| M032 | 1914 | SVIC | historic | -37.876, 145.095 | Ashburton | M | TOE PAD | N/A | American Museum of Natural History |
| M033 | 1914 | SVIC | historic | -37.876, 145.095 | Ashburton | M | TOE PAD | N/A | American Museum of Natural History |
| M034 | 1914 | SVIC | historic | -37.876, 145.095 | Ashburton | M | TOE PAD | N/A | American Museum of Natural History |
| M035 | 1914 | SVIC | historic | -37.876, 145.095 | Ashburton | F | TOE PAD | N/A | American Museum of Natural History |
| M036 | 1914 | SVIC | historic | -37.876, 145.095 | Ashburton | F | TOE PAD | N/A | American Museum of Natural History |
| M037 | 1914 | SVIC | historic | -37.876, 145.095 | Ashburton | F | TOE PAD | N/A | American Museum of Natural History |
| M038 | 1898 | SVIC | historic | -38.276, 145.595 | Lang Lang | M | TOE PAD | N/A | American Museum of Natural History |
| M039 | 1895 | SVIC | historic | -38.076, 144.295 | Moorabool | U | TOE PAD | N/A | American Museum of Natural History |
| M040 | 1908 | SVIC | historic | -37.916, 145.155 | Mulgrave | M | TOE PAD | N/A | American Museum of Natural History |
| M041 | 1898 | ACT | historic | -N/A | N/A | F | TOE PAD | N/A | American Museum of Natural History |
| M042 | 1966 | QLD | historic | -28.23, 151.75 | Greymare | M | TOE PAD | N/A | Queensland Museum |
| M045 | N/A | QLD | historic | -26.74, 150.65 | Chinchilla | M | TOE PAD | N/A | Queensland Museum |
| M047 | 1919 | ADL | historic | -35.050, 138.7 | Clarendon, | M | TOE PAD | N/A | South Australia Museum |
| M048 | 1919 | ADL | historic | -35.150, 138.6 | Happy Valley | F | TOE PAD | N/A | South Australia Museum |
| M049 | 1917 | ADL | historic | -35.0290, 138.6 | Blackwood | M | TOE PAD | N/A | South Australia Museum |
| M050 | 1926 | ADL | historic | -35.69, 137.59 | Kangaroo Island | M | TOE PAD | N/A | South Australia Museum |
| M051 | 1918 | ADL | historic | -35.0290, 138.6 | Blackwood | M | TOE PAD | N/A | South Australia Museum |
| M052 | 1918 | ADL | historic | -35.0290, 138.6 | Blackwood | F | TOE PAD | N/A | South Australia Museum |
| M053 | NA | ADL | historic | -34.60, 138.75 | Gawler | U | TOE PAD | N/A | South Australia Museum |
| M054 | 1914 | ADL | historic | -35.0605, 138.68 | Cherry Gardens | M | TOE PAD | N/A | South Australia Museum |
| M055 | NA | NVIC | historic | -35.75, 147.29 | Holbrook | U | TOE PAD | N/A | South Australia Museum |
| M056 | 1936 | ADL | historic | -35.0605, 138.59 | Coromandel Valley | F | TOE PAD | N/A | South Australia Museum |
| M057 | NA | ADL | historic | -35.380, 138.63 | Square waterhole | M | TOE PAD | N/A | South Australia Museum |
| M058 | 1894 | BMTN | historic | -33.790, 150.941 | Toongabbie | F | TOE PAD | N/A | South Australia Museum |
| M059 | 1900 | BMTN | historic | -33.85, 151.13 | Abbotsford | M | TOE PAD | N/A | Museum Victoria |
| M060 | 1906 | BMTN | historic | -33.92, 151.03 | Bankstown | M | TOE PAD | N/A | Museum Victoria |
| M061 | 1909 | SVIC | historic | -37.65, 145.52 | Healesville | M | TOE PAD | N/A | Museum Victoria |
| M062 | 1903 | SVIC | historic | -37.72, 145.15 | Eltham | M | TOE PAD | N/A | Museum Victoria |
| M063 | 1907 | SVIC | historic | -37.05, 144.8 | Tooborac | F | TOE PAD | N/A | Museum Victoria |
| M064 | 1905 | SVIC | historic | -37.52, 145.13 | Whittlesea | M | TOE PAD | N/A | Museum Victoria |
| M065 | 1931 | NVIC | historic | -36.47, 147.25 | Eskdale district | U | TOE PAD | N/A | Museum Victoria |
| M067 | 1917 | BMTN | historic | -34.05, 151.15 | Sutherland shire | F | TOE PAD | N/A | Australian Museum |
| M072 | 1912 | BMTN | historic | -33.783, 150.95 | Parramatta | M | TOE PAD | N/A | Australian Museum |
| R041 | 1996 | BMTN | recent | -33.08327, 150.18502 | Capertee** | M | BLOOD | 7-rhe orange | Museum Victoria |
| R050 | 1996 | BMTN | recent | -32.4, 149.87 | Cumbo Road | F | BLOOD | 041-57207 | Museum Victoria |
| R073 | 1997 | NNSW | recent | -30.3, 150.79 | Armidale | F | BLOOD | 042-03907 | Museum Victoria |
| N009 | 2015 | BMTN | new | -33.01383, 150.03304 | Capertee | M | BLOOD | 053-11099 | Australian National University |
| R001 | 1995 | NNSW | recent | -30.435, 151.225 | Armidale | U | BLOOD | 041-48942 | Museum Victoria |
| R002 | 1995 | NNSW | recent | -30.43, 151.22 | Armidale | U | BLOOD | 041-48943 | Museum Victoria |
| R003 | 1996 | NNSW | recent | -30.43, 151.22 | Armidale | M | BLOOD | 041-48945 | Museum Victoria |
| R004 | 1996 | NNSW | recent | -30.43, 151.22 | Armidale | M | BLOOD | 041-48946 | Museum Victoria |
| R005 | 1995 | NNSW | recent | -30.436, 151.217 | Armidale | U | BLOOD | 041-48951 | Museum Victoria |
| R006 | 1995 | NNSW | recent | -30.43, 151.22 | Armidale | F | BLOOD | 041-48952 | Museum Victoria |
| R007 | 1995 | NNSW | recent | -30.43, 151.22 | Armidale | M | BLOOD | 041-48953 | Museum Victoria |
| R008 | 1996 | NNSW | recent | -30.43, 151.22 | Armidale | M | BLOOD | 041-48985 | Museum Victoria |
| R009 | 1995 | BMTN | recent | -33.04627, 150.16257 | Capertee | M | BLOOD | 041-57159 | Museum Victoria |
| R010 | 1995 | BMTN | recent | -33.04627, 150.16257 | Capertee | M | BLOOD | 041-57161 | Museum Victoria |
| R011 | 1995 | BMTN | recent | -33.04627, 150.16257 | Capertee | M | BLOOD | 041-57162 | Museum Victoria |
| R012 | 1995 | BMTN | recent | -33.04627, 150.16257 | Capertee | M | BLOOD | 041-57163 | Museum Victoria |
| R013 | 1995 | BMTN | recent | -33.04627, 150.16257 | Capertee | F | BLOOD | 041-57164 | Museum Victoria |
| R014 | 1995 | BMTN | recent | -33.04627, 150.16257 | Capertee | M | BLOOD | 041-57165 | Museum Victoria |
| R015 | 1995 | BMTN | recent | -33.04627, 150.16257 | Capertee | M | BLOOD | 041-57166 | Museum Victoria |
| R016 | 1995 | BMTN | recent | -33.04627, 150.16257 | Capertee | M | BLOOD | 041-57167 | Museum Victoria |
| R018 | 1995 | BMTN | recent | -33.04627, 150.16257 | Capertee | M | BLOOD | 041-57169 | Museum Victoria |
| R019 | 1995 | BMTN | recent | -32.95627, 150.10257 | Capertee | M | BLOOD | 041-57170 | Museum Victoria |
| R020 | 1995 | BMTN | recent | -32.95627, 150.10257 | Capertee | F | BLOOD | 041-57171 | Museum Victoria |
| R021 | 1995 | BMTN | recent | -32.95627, 150.10257 | Capertee | M | BLOOD | 041-57173 | Museum Victoria |
| R022 | 1995 | BMTN | recent | -33.01756, 150.03326 | Capertee | F | BLOOD | 041-57176 | Museum Victoria |
| R023 | 1995 | BMTN | recent | -33.01756, 150.03326 | Capertee | M | BLOOD | 041-57177 | Museum Victoria |
| R024 | 1995 | BMTN | recent | -33.01756, 150.03326 | Capertee | F | BLOOD | 041-57178 | Museum Victoria |
| R025 | 1995 | BMTN | recent | -33.01756, 150.03326 | Capertee | M | BLOOD | 041-57179 | Museum Victoria |
| R026 | 1995 | BMTN | recent | -33.01756, 150.03326 | Capertee | M | BLOOD | 041-57180 | Museum Victoria |
| R027 | 1995 | BMTN | recent | -33.01756, 150.03326 | Capertee | M | BLOOD | 041-57181 | Museum Victoria |
| R028 | 1995 | NVIC | recent | -36.15, 146.65 | Chiltern | U | BLOOD | 041-87302 | Museum Victoria |
| R029 | 1995 | NVIC | recent | -36.154, 146.645 | Chiltern | U | BLOOD | 041-87303 | Museum Victoria |
| R030 | 1995 | NVIC | recent | -36.15, 146.65 | Chiltern | U | BLOOD | 041-87305 | Museum Victoria |
| R031* | 1995 | NVIC | recent | -36.158, 146.605 | Chiltern | U | BLOOD | 041-87306 | Museum Victoria |
| R032 | 1995 | ACT | recent | -35.265, 149.175 | Canberra | F | BLOOD | 041-87307 | Museum Victoria |
| R033 | 1995 | ACT | recent | -35.265, 149.175 | Canberra | M | BLOOD | 041-87308 | Museum Victoria |
| R034 | 1995 | ACT | recent | -35.265, 149.175 | Canberra | M | BLOOD | 041-87309 | Museum Victoria |
| R035 | 1995 | ACT | recent | -35.265, 149.175 | Canberra | F | BLOOD | 041-87310 | Museum Victoria |
| R036 | 1995 | ACT | recent | -35.265, 149.175 | Canberra | M | BLOOD | 041-87311 | Museum Victoria |
| R037 | 1995 | ACT | recent | -35.265, 149.175 | Canberra | F | BLOOD | 041-87312 | Museum Victoria |
| R038* | 1995 | ACT | recent | -35.265, 149.175 | Canberra | M | BLOOD | 041-87313 | Museum Victoria |
| R039 | 1996 | BMTN | recent | -33.08327, 150.185025 | Capertee** | F | BLOOD | 2-rhe yellow | Museum Victoria |
| R040 | 1996 | BMTN | recent | -33.08327, 150.185025 | Capertee** | F | BLOOD | 4-rhe white | Museum Victoria |
| R042 | 1996 | BMTN | recent | -33.08327, 150.185025 | Capertee** | M | BLOOD | 8-rhe yellow/orange | Museum Victoria |
| R043 | 1996 | BMTN | recent | -33.08327, 150.185025 | Capertee** | F | BLOOD | 9-rhe black | Museum Victoria |
| R044 | 1996 | BMTN | recent | -36.2, 146.7 | Chiltern** | M | BLOOD | 1-rhe red | Museum Victoria |
| R045 | 1996 | BMTN | recent | -36.2, 146.7 | Chiltern** | F | BLOOD | 3-rhe blue | Museum Victoria |
| R046 | 1996 | BMTN | recent | -36.2, 146.7 | Chiltern** | F | BLOOD | 5-rhe purple | Museum Victoria |
| R047 | 1996 | BMTN | recent | -36.2, 146.7 | Chiltern** | M | BLOOD | 6-rhe light green | Museum Victoria |
| R048 | 1996 | BMTN | recent | -32.4, 149.87 | Cumbo Rd, NSW | F | BLOOD | 041-57204 | Museum Victoria |
| R049 | 1996 | BMTN | recent | -32.4, 149.87 | Cumbo Rd, NSW | M | BLOOD | 041-57206 | Museum Victoria |
| R051 | 1996 | BMTN | recent | -32.25, 150.05 | Goulburn River NP | M | BLOOD | 041-57208 | Museum Victoria |
| R052 | 1996 | BMTN | recent | -32.25, 150.05 | Goulburn River NP | M | BLOOD | 041-57209 | Museum Victoria |
| R053 | 1996 | BMTN | recent | -32.389, 149.835 | Munghorn East | M | BLOOD | 041-57205 | Museum Victoria |
| R054 | 1996 | NNSW | recent | -30.43, 151.22 | Armidale | M | BLOOD | 041-48948 | Museum Victoria |
| R055 | 1995 | BMTN | recent | -33.08327, 150.185025 | Capertee | M | BLOOD | 041-57172 | Museum Victoria |
| R056 | 1995 | BMTN | recent | -33.08327, 150.185025 | Capertee | U | BLOOD | 041-57183 | Museum Victoria |
| R057 | 1997 | NVIC | recent | -36.15, 146.65 | Chiltern | U | BLOOD | 041-87401 | Museum Victoria |
| R058 | 1997 | NVIC | recent | -36.15, 146.65 | Chiltern | M | BLOOD | 041-87402 | Museum Victoria |
| R059 | 1997 | NVIC | recent | -36.15, 146.65 | Chiltern | M | BLOOD | 041-87403 | Museum Victoria |
| R060 | 1997 | NVIC | recent | -36.15, 146.65 | Chiltern | U | BLOOD | 041-87404 | Museum Victoria |
| R061 | 1997 | NVIC | recent | -36.15, 146.65 | Chiltern | M | BLOOD | 041-87405 | Museum Victoria |
| R062 | 1997 | NVIC | recent | -36.15, 146.65 | Chiltern | U | BLOOD | 041-87406 | Museum Victoria |
| R063 | 1997 | NVIC | recent | -36.15, 146.65 | Chiltern | F | BLOOD | 041-87407 | Museum Victoria |
| R064 | 1997 | NNSW | recent | -30.43, 151.22 | Armidale | M | BLOOD | 042-03910 | Museum Victoria |
| R065 | 1995 | NNSW | recent | -30.43, 151.22 | Armidale | U | BLOOD | 041-48912 | Museum Victoria |
| R066 | 1996 | NNSW | recent | -30.43, 151.22 | Armidale | M | BLOOD | 041-48983 | Museum Victoria |
| R067 | 1997 | NNSW | recent | -30.415, 151.165 | Armidale | F | BLOOD | 041-48986 | Museum Victoria |
| R068 | 1997 | NNSW | recent | -30.43, 151.22 | Armidale | F | BLOOD | 041-48987 | Museum Victoria |
| R069 | 1995 | NVIC | recent | -36.15, 146.65 | Chiltern | U | BLOOD | 041-87301 | Museum Victoria |
| R070 | 1995 | NVIC | recent | -36.15, 146.65 | Chiltern | U | BLOOD | 041-87304 | Museum Victoria |
| R071 | 1997 | NNSW | recent | -30.3, 150.79 | Armidale | M | BLOOD | 042-03901 | Museum Victoria |
| R072 | 1997 | NNSW | recent | -30.3, 150.79 | Armidale | U | BLOOD | 042-03902 | Museum Victoria |
| R074 | 1997 | NNSW | recent | -30.3, 150.79 | Armidale | M | BLOOD | 042-03908 | Museum Victoria |
| R076 | 1997 | NNSW | recent | -30.3, 150.79 | Armidale | M | BLOOD | 042-03911 | Museum Victoria |
| R077 | 1997 | NNSW | recent | -30.3, 150.79 | Armidale | U | BLOOD | 042-03912 | Museum Victoria |
| R078 | 1997 | NNSW | recent | -30.3, 150.79 | Armidale | F | BLOOD | 042-03914 | Museum Victoria |
| R079 | 1997 | NNSW | recent | -30.3, 150.79 | Armidale | M | BLOOD | 042-03915 | Museum Victoria |
| R080 | 1989 | ACT | recent | -35.185, 149.25 | Sutton | F | BLOOD | f724 | Museum Victoria |
| R081 | 1989 | ACT | recent | -35.185, 149.25 | Sutton | M | BLOOD | f725 | Museum Victoria |
| R082 | 2011 | BMTN | recent | -33.15327, 150.135025 | Capertee | F | BLOOD | 042-99755 | Museum Victoria |
| R083 | 2011 | BMTN | recent | -33.15327, 150.135025 | Capertee | M | BLOOD | 042-99757 | Museum Victoria |
| R084 | 2011 | BMTN | recent | -33.15327, 150.135025 | Capertee | M | BLOOD | 042-99703 | Museum Victoria |
| R085 | 2011 | BMTN | recent | -33.15327, 150.135025 | Capertee | M | BLOOD | 042-99704 | Museum Victoria |
| R086 | 2011 | BMTN | recent | -33.15327, 150.135025 | Capertee | F | BLOOD | 042-99753 | Museum Victoria |
| R087 | 2011 | NVIC | recent | -36.18, 146.775 | Indigo Valley | M | BLOOD | 042-99754 | Museum Victoria |
| R088 | 2011 | NVIC | recent | -36.6, 146.1 | Lurg | M | BLOOD | 042-99705 | Museum Victoria |
| R089 | 2012 | BMTN | recent | -33.10327, 150.205025 | Capertee | M | BLOOD | 042-99767 | Museum Victoria |
| R090 | 2012 | BMTN | recent | -33.10327, 150.205025 | Capertee | M | BLOOD | 042-99768 | Museum Victoria |
| R091 | 2012 | BMTN | recent | -33.11127, 150.265025 | Capertee | M | BLOOD | 042-99769 | Museum Victoria |
| R092 | 2012 | BMTN | recent | -33.11127, 150.265025 | Capertee | M | BLOOD | 042-99773 | Museum Victoria |
| R093 | 2012 | BMTN | recent | -33.11127, 150.265025 | Capertee | M | BLOOD | 042-99774 | Museum Victoria |
| R094 | 2012 | BMTN | recent | -33.11127, 150.265025 | Capertee | M | BLOOD | 042-99775 | Museum Victoria |
| R095 | 2012 | BMTN | recent | -33.11127, 150.265025 | Capertee | F | BLOOD | 042-99776 | Museum Victoria |
| R096 | 2012 | BMTN | recent | -33.11127, 150.265025 | Capertee | M | BLOOD | 042-99777 | Museum Victoria |
| R097 | 2012 | BMTN | recent | -32.915, 151.25 | Quorrobolong | M | BLOOD | 042-99758 | Museum Victoria |
| R098 | 2012 | BMTN | recent | -32.915, 151.25 | Quorrobolong | M | BLOOD | 042-99759 | Museum Victoria |
| R099 | 2012 | BMTN | recent | -32.915, 151.25 | Quorrobolong | M | BLOOD | 042-99761 | Museum Victoria |
| R100 | 2012 | BMTN | recent | -32.915, 151.25 | Quorrobolong | M | BLOOD | 042-99762 | Museum Victoria |
| R101 | 2012 | BMTN | recent | -32.915, 151.25 | Quorrobolong | F | BLOOD | 042-99763 | Museum Victoria |
| R102 | 2012 | BMTN | recent | -32.915, 151.25 | Quorrobolong | M | BLOOD | 042-99764 | Museum Victoria |
| R103 | 2012 | BMTN | recent | -32.915, 151.25 | Quorrobolong | F | BLOOD | 042-99765 | Museum Victoria |
| R104 | 2012 | BMTN | recent | -32.915, 151.25 | Quorrobolong | M | BLOOD | 042-99766 | Museum Victoria |
| * Denotes sample included in ddRAD | | | | | | | | | |
