# Supplementary material for: Genomic impact of severe population decline in a nomadic songbird
Source: PLoS One. 2019 Oct 24;14(10):e0223953. doi: 10.1371/journal.pone.0223953 (PMC6812763; doi:10.1371/journal.pone.0223953)
Supplement: S2 Table — (DOCX) [file pone.0223953.s002.docx]

| **PERIOD** | **POPULATION** | **MinDP15, maxDP100, no cutoff** | | **minDP15, maxDP100 cutoff = 0.65** | | **DP15, no cutoff** | | **DP10, no cutoff** | | **DP5, no cutoff** | | **DP5, missingno cutoff = 0.65** | |
| --- | --- | --- | --- | --- | --- | --- | --- | --- | --- | --- | --- | --- | --- |
|  |  | Number | % | Number | % | Number | % | Number | % | Number | % | Number | % |
| ALL | HISTORIC | 0 | N/A | 0 | N/A | 0 | N/A | 0 | N/A | 0 | 0 | 0 | 0 |
|  | RECENT | 0 | N/A | 0 | N/A | 0 | N/A | 0 | N/A | 2 | 100 | 2 | 100 |
|  | CURRENT | 0 | N/A | 0 | N/A | 0 | N/A | 0 | N/A | 0 | 0 | 0 | 0 |
|  |  |  |  |  |  |  |  |  |  |  |  |  |  |
| HISTORIC | ADL | 9 | 11 | 2 | 14 | 12 | 16 | 24 | 17 | 131 | 18 | 33 | 20 |
|  | SVIC | 45 | 54 | 7 | 50 | 47 | 61 | 71 | 50 | 440 | 58 | 102 | 61 |
|  | NVIC | 2 | 3 | 1 | 7 | 2 | 3 | 9 | 6 | 21 | 3 | 6 | 4 |
|  | BMTN | 7 | 9 | 3 | 22 | 7 | 9 | 14 | 10 | 74 | 10 | 23 | 14 |
|  | NNSW | 10 | 12 | 1 | 7 | 9 | 12 | 24 | 17 | 78 | 10 | 3 | 1 |
|  |  |  |  |  |  |  |  |  |  |  |  |  |  |
| RECENT | recentBMTN | 0 | 0 | 0 | 0 | 0 | 0 | 0 | 0 | 0 | 0 | 0 | 0 |
|  | recentNNSW | 0 | 0 | 0 | 0 | 0 | 0 | 0 | 0 | 0 | 0 | 0 | 0 |
|  | recentNVIC | 0 | 0 | 0 | 0 | 0 | 0 | 0 | 0 | 0 | 0 | 0 | 0 |
| CURRENT | currentBMTN | 1 | 100 | 1 | 100 | 1 | 100 | 3 | 100 | 1 | 50 | 1 | 50 |
|  | currentNNSW | 0 | 0 | 0 | 0 | 0 | 0 | 0 | 0 | 1 | 50 | 1 | 50 |
