# Supplementary material for: Genomic impact of severe population decline in a nomadic songbird
Source: PLoS One. 2019 Oct 24;14(10):e0223953. doi: 10.1371/journal.pone.0223953 (PMC6812763; doi:10.1371/journal.pone.0223953)
Supplement: S1 Fig — (A) ddRAD libraries and (B) whole genome libraries, hybridization, and sequencing. (PDF) [file pone.0223953.s003.pdf]

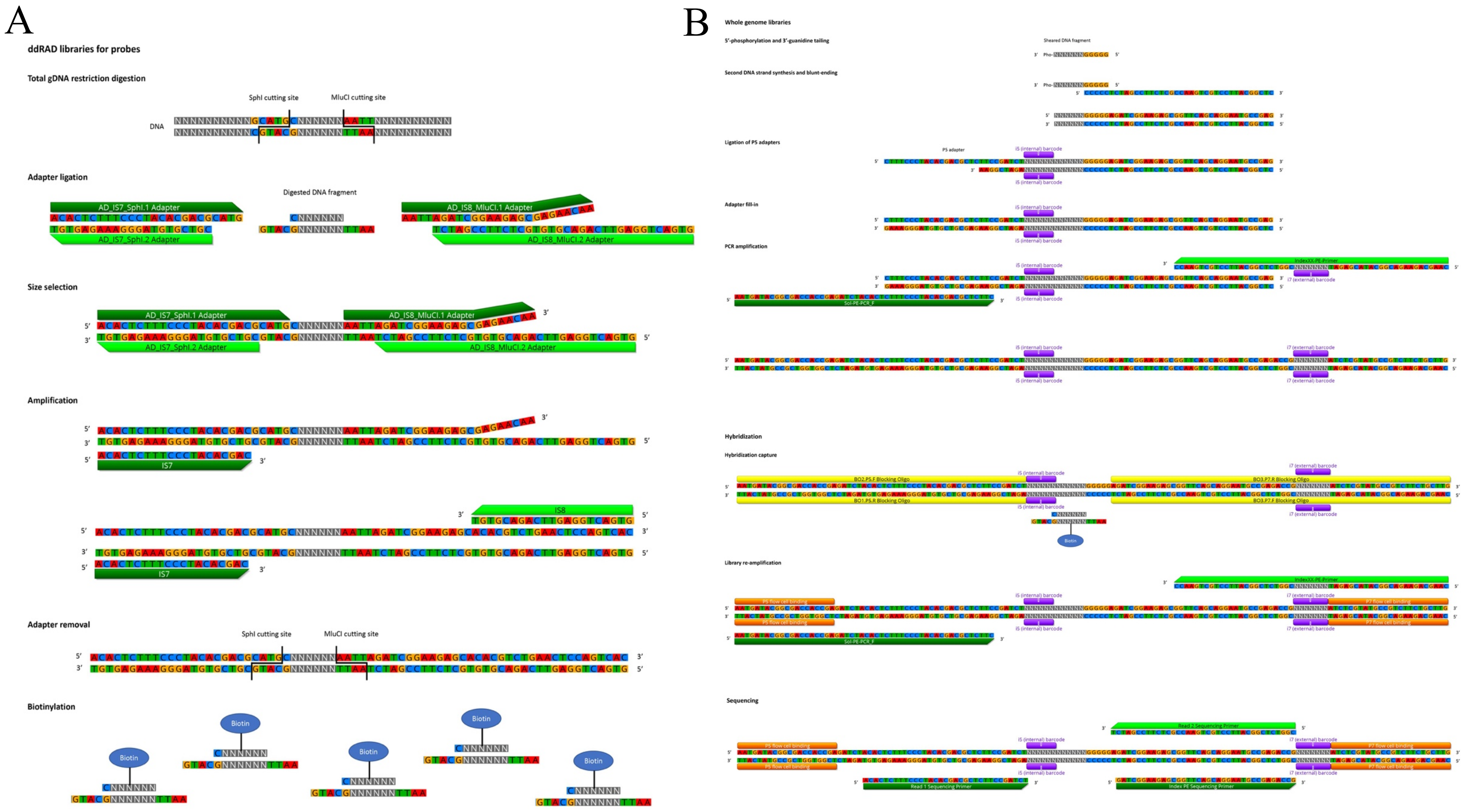
A
ddRAD libraries for probes
Total gDNA restriction digestion
SphI cutting site
MluCI cutting site
DNA
Adapter ligation
Digested DNA fragment
AD_IS7_SphI.1 Adapter
AD_IS7_SphI.2 Adapter
AD_IS8_MluCI.1 Adapter
AD_IS8_MluCI.2 Adapter
Size selection
Amplification
IS7
IS8
Adapter removal
Biotinylation
Biotin
B
Whole genome libraries
5'-phosphorylation and 3'-guanidine tailing
Sheared DNA fragment
Second DNA strand synthesis and blunt-ending
Ligation of P5 adapters
P5 adapter
i5 (internal) barcode
Adapter fill-in
PCR amplification
IndexXX-PE-Primer
i7 (external) barcode
Sol-PE-PCR_F
Hybridization
Hybridization capture
BO2.P5.F Blocking Oligo
BO3.P7.R Blocking Oligo
BO1.P5.R Blocking Oligo
BO3.P7.F Blocking Oligo
Library re-amplification
P5 flow cell binding
P7 flow cell binding
Sequencing
Read 2 Sequencing Primer
Read 1 Sequencing Primer
Index PE Sequencing Primer
