# Supplementary figures and images for: Genomic impact of severe population decline in a nomadic songbird
Source: PLoS One. 2019 Oct 24;14(10):e0223953. doi: 10.1371/journal.pone.0223953 (PMC6812763; doi:10.1371/journal.pone.0223953)

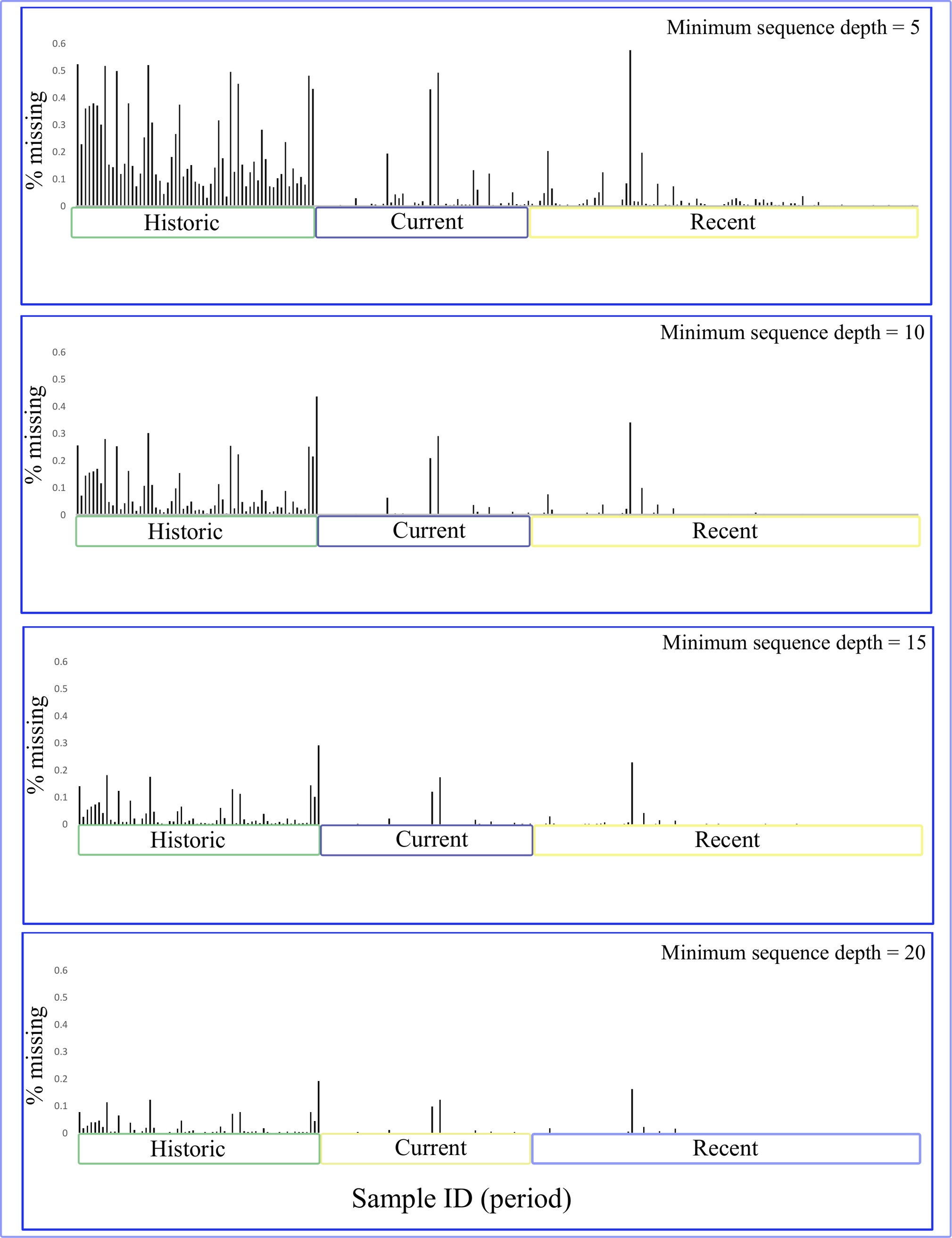

Supplement: S2 Fig — (TIF) [file pone.0223953.s004.tif]

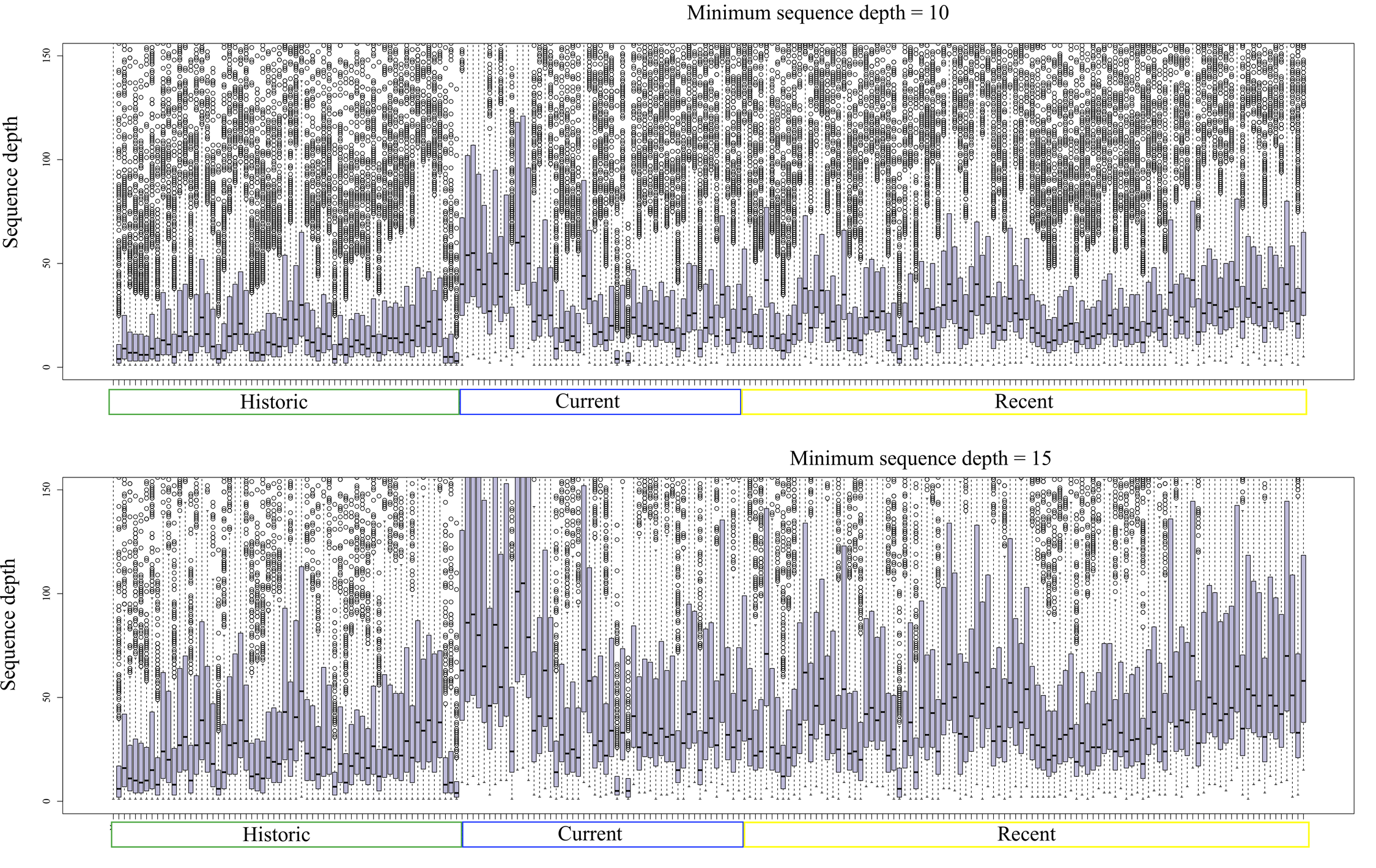

Supplement: S3 Fig — Boxplots of sequence depth by sample and sampling period in the regent honeyeater hyRAD dataset at minimum read depths of (A) 10 and (B) 15. (TIF) [file pone.0223953.s005.tif]

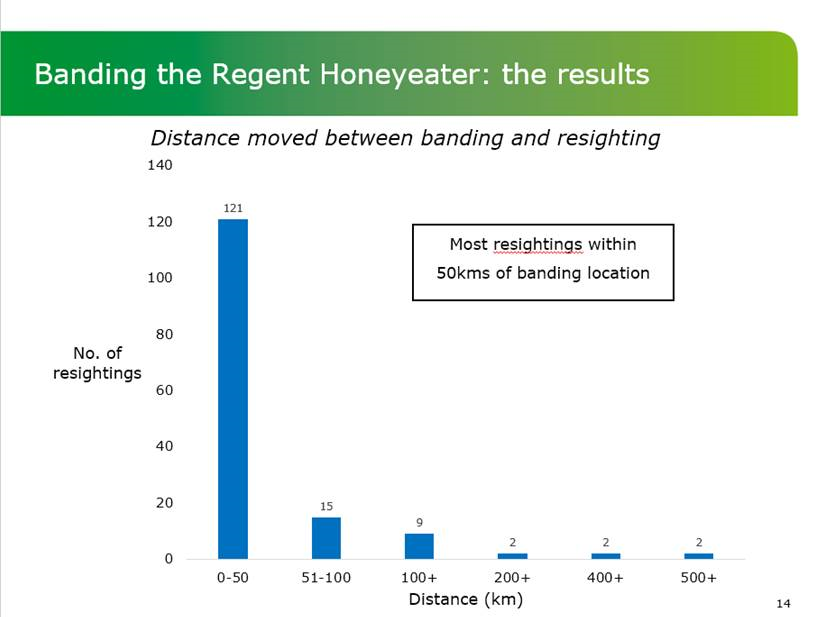

Supplement: S4 Fig — Data collected from BirdLife Australia citizen science project (D.Ingwersen, unpublished data). Distances calculated using the measure tool in GoogleEarth. (TIF) [file pone.0223953.s006.tif]

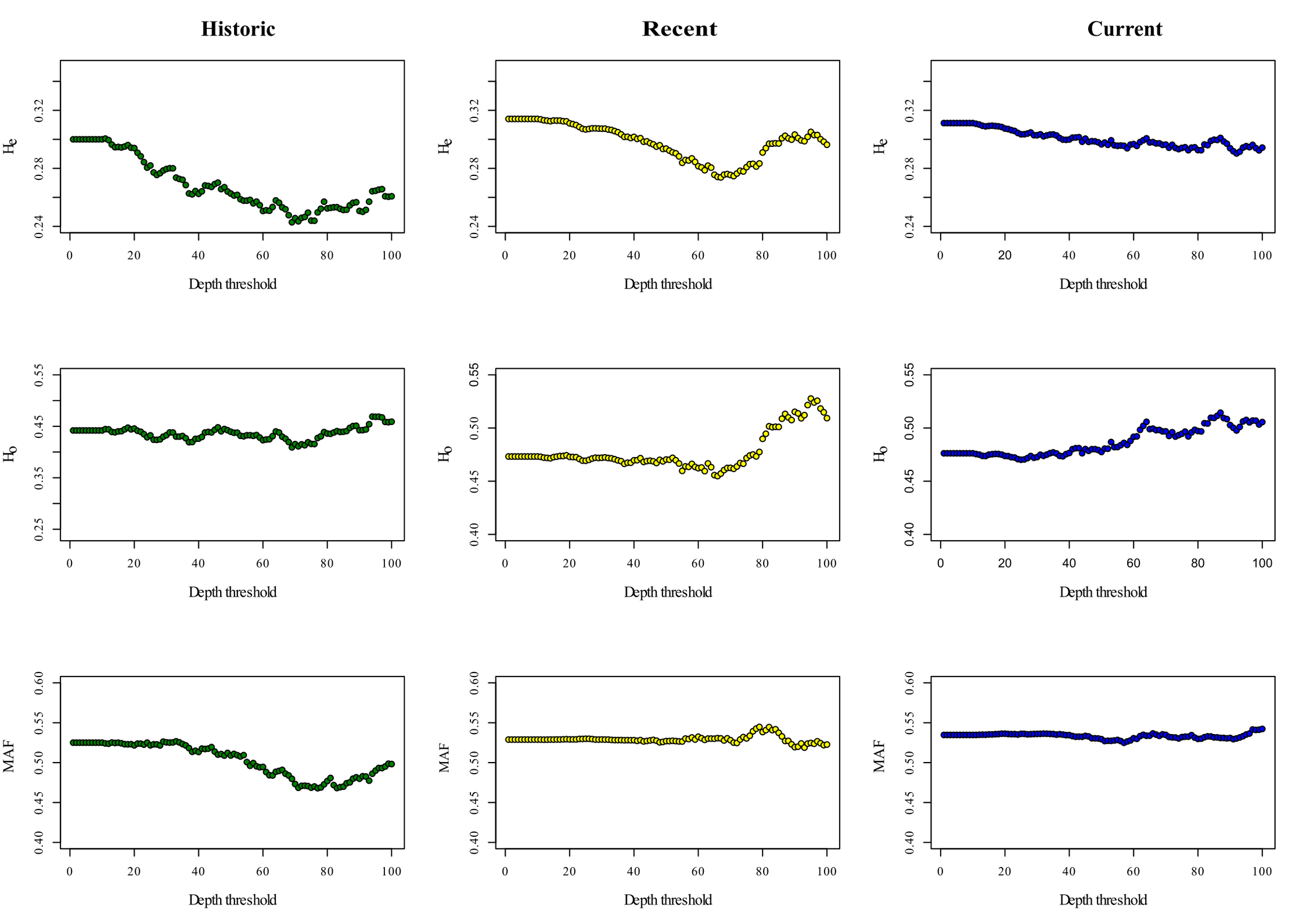

Supplement: S5 Fig — (TIF) [file pone.0223953.s007.tif]

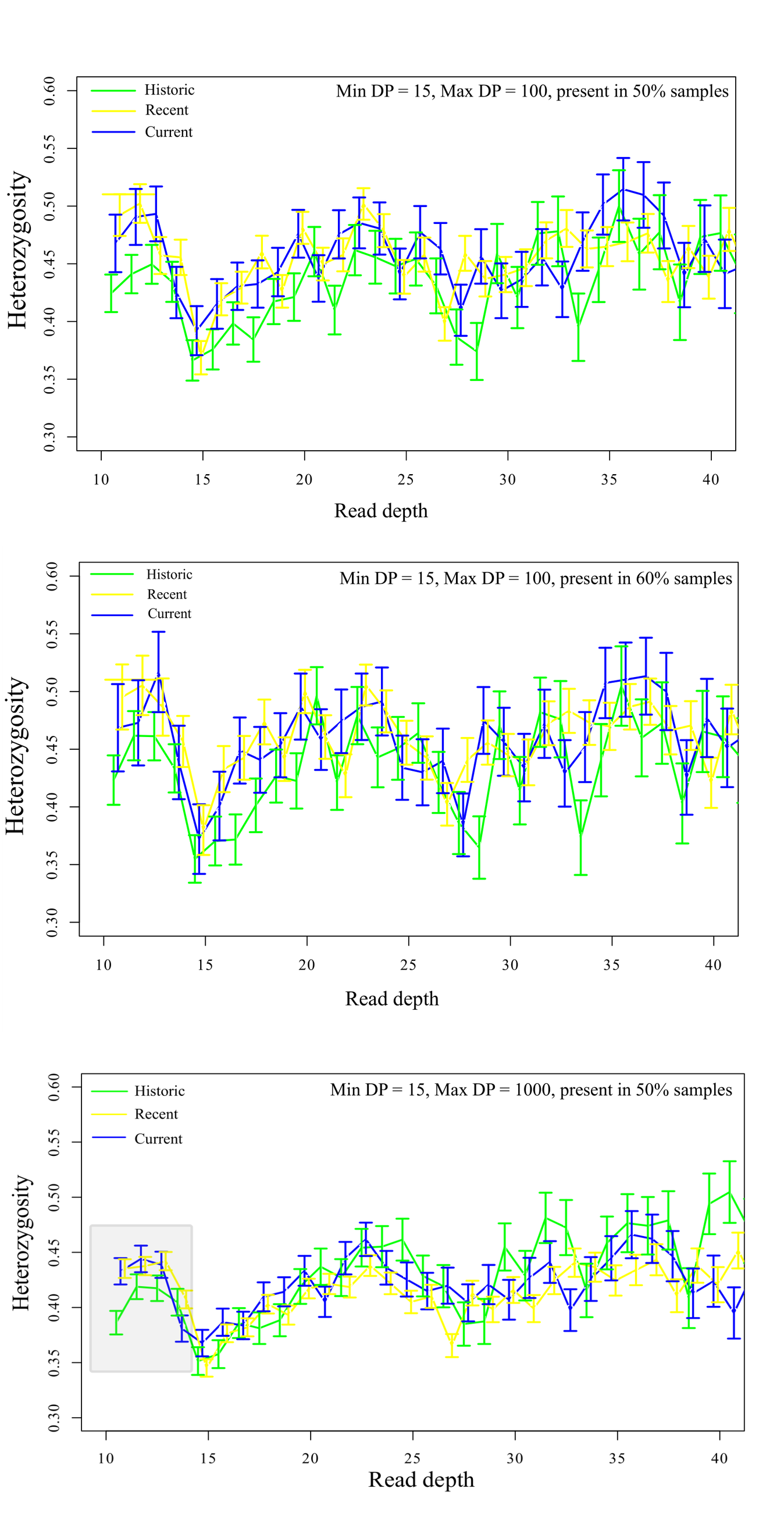

Supplement: S6 Fig — Highlighted area shows significantly lower heterozygosity in the historic sample at read depths < 13, hence the use of min DP = 15 for temporal genetic diversity analyses. (TIF) [file pone.0223953.s008.tif]

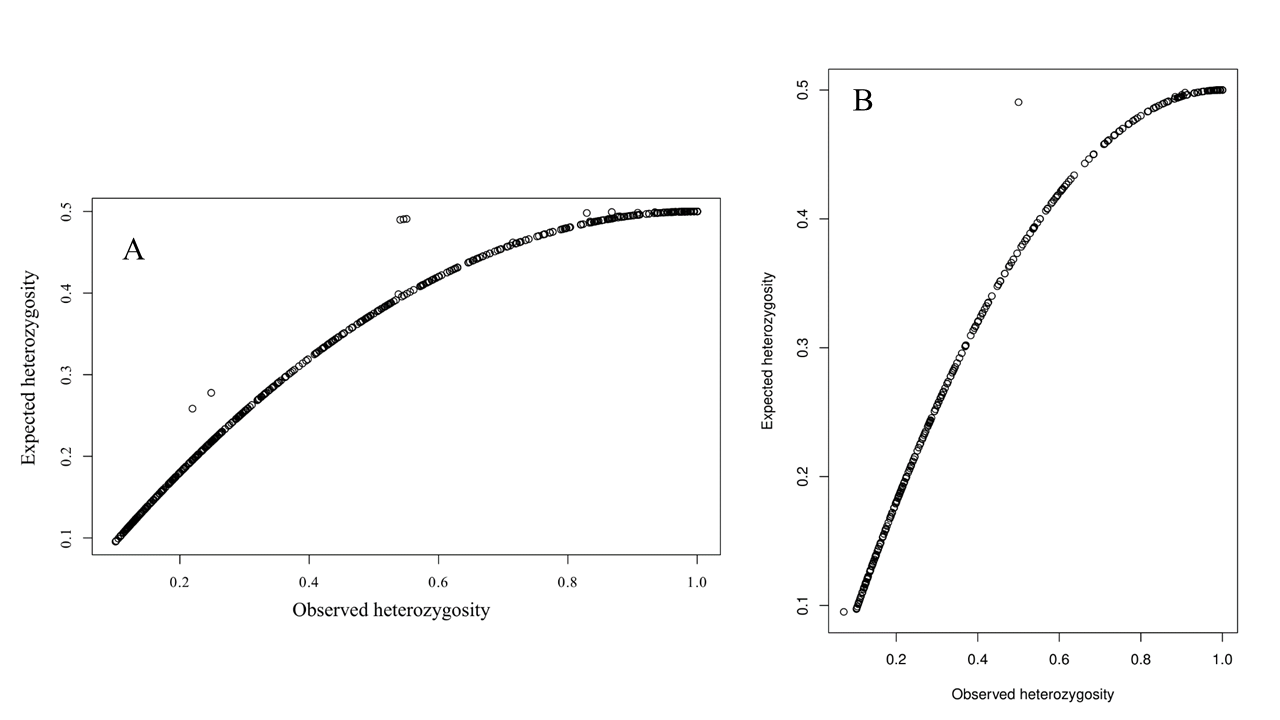

Supplement: S7 Fig — (A): Minimum sequence depth = 10, missingness. cutoff = 0.75. (B): Minimum sequence depth 15, maximum sequence depth 100, missingness. cutoff = 0.65. (TIF) [file pone.0223953.s009.tif]

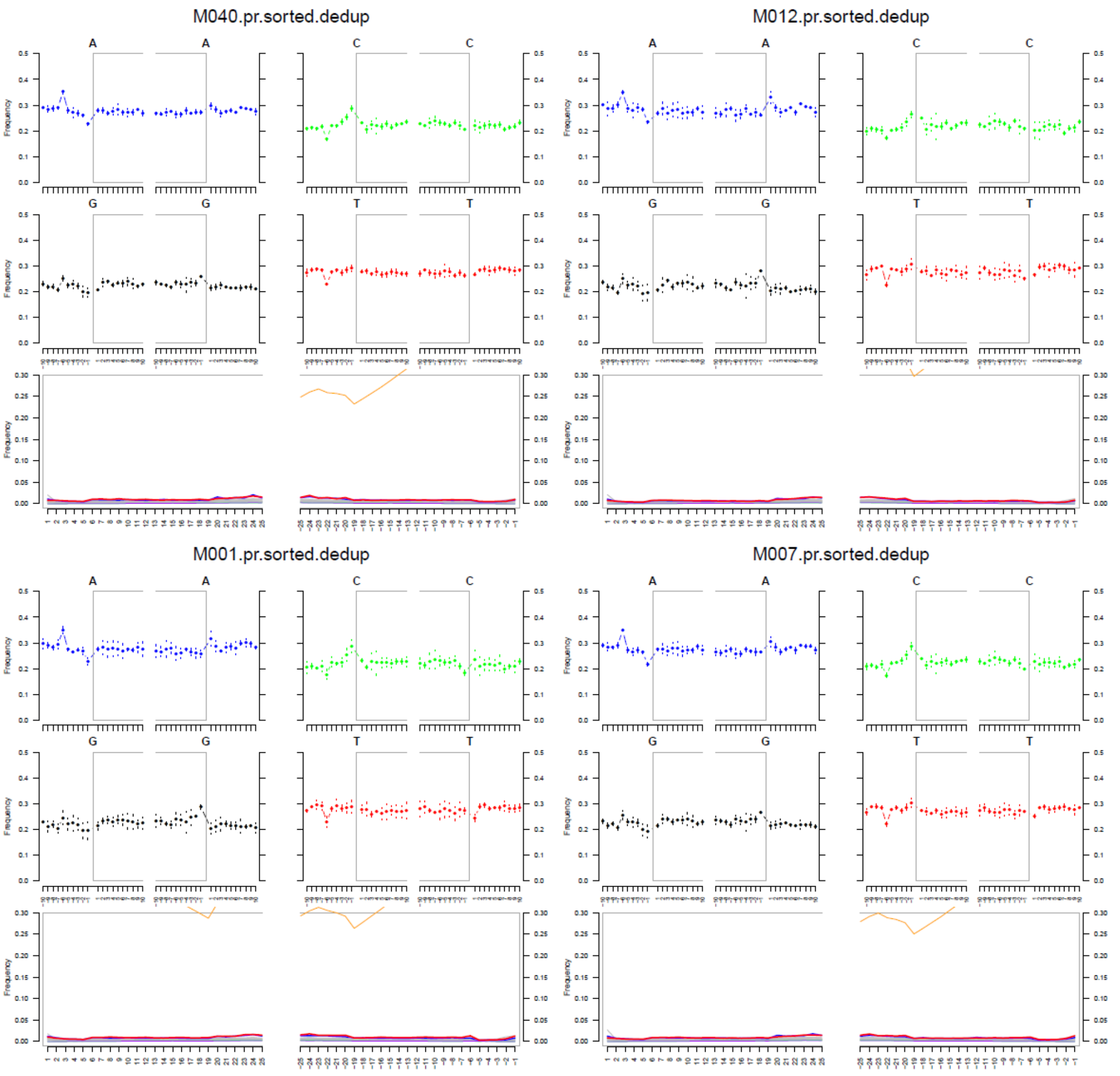

Supplement: S8 Fig — (TIFF) [file pone.0223953.s010.tiff]

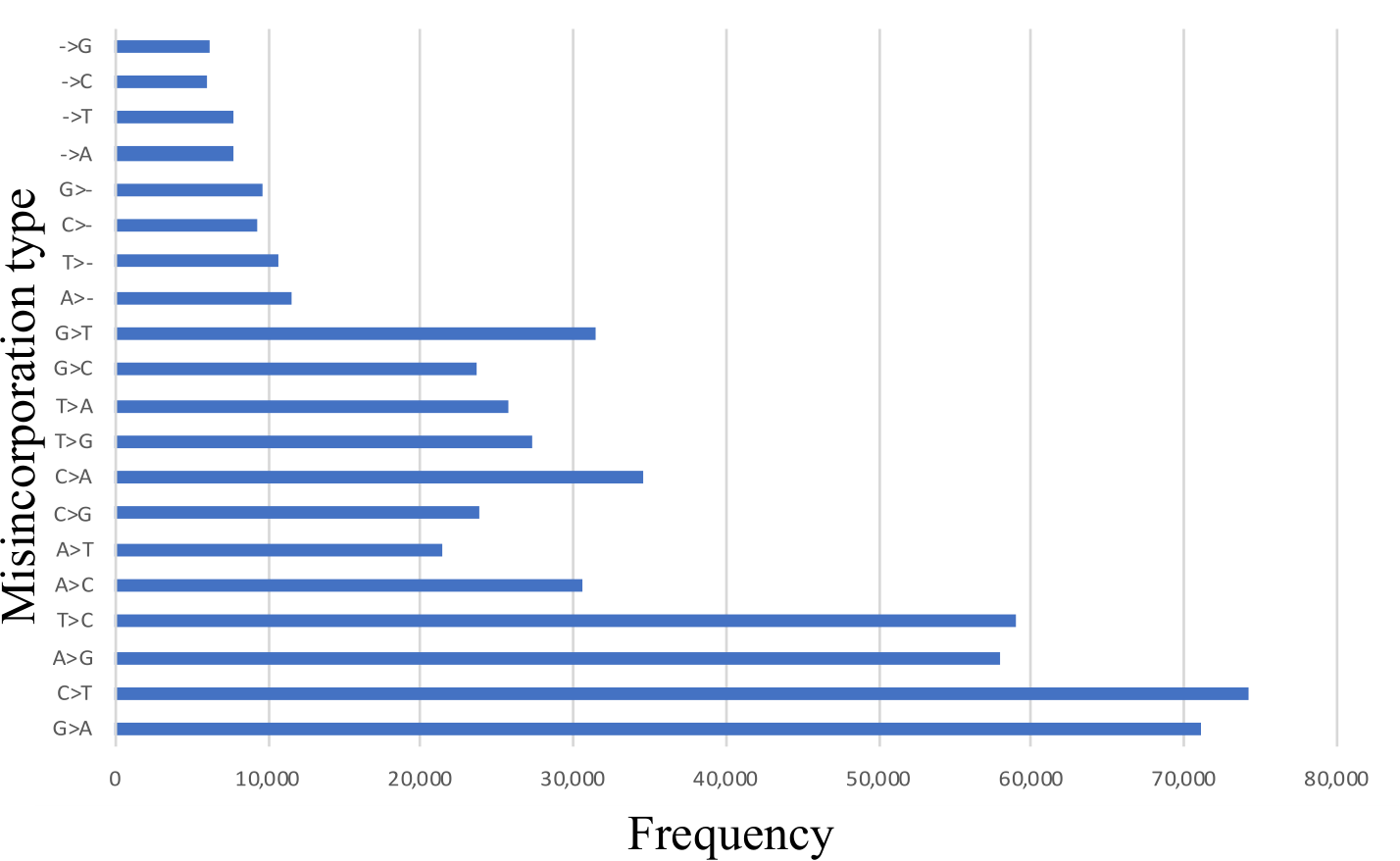

Supplement: S9 Fig — (TIF) [file pone.0223953.s011.tif]

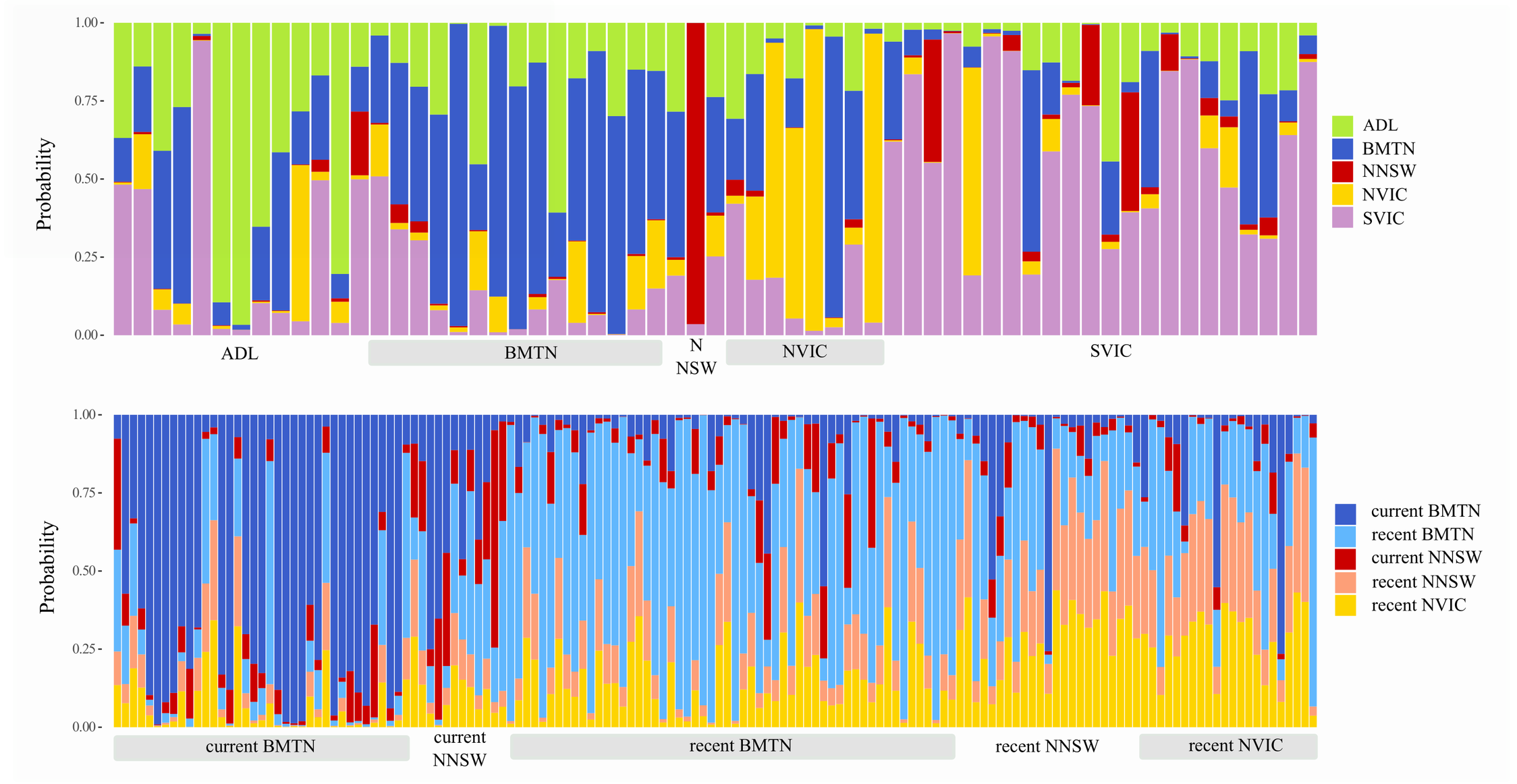

Supplement: S10 Fig — (TIF) [file pone.0223953.s012.tif]

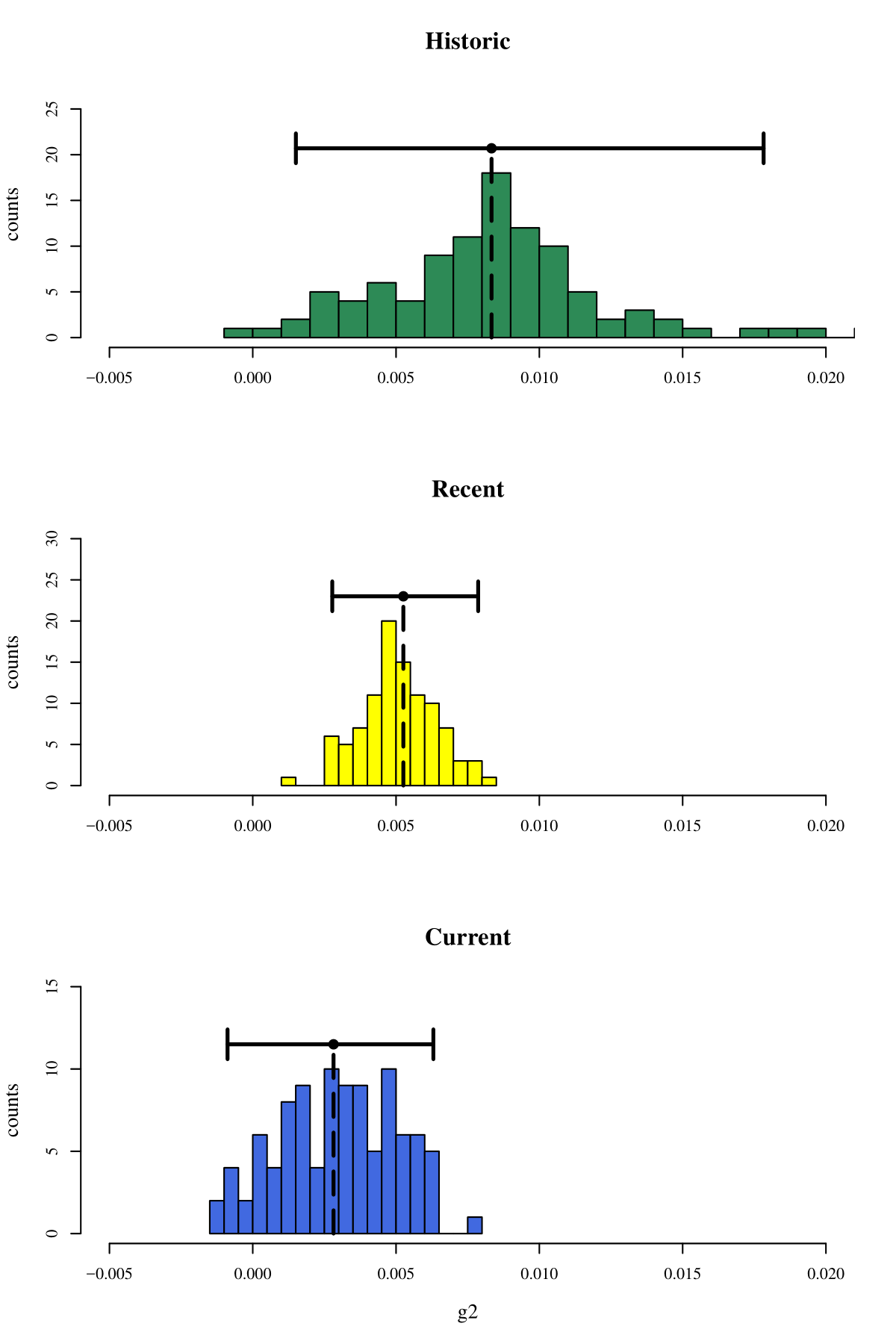

Supplement: S12 Fig — Lateral error bars represent 95% confidence intervals estimated via 100 bootstraps of each sample. (TIF) [file pone.0223953.s014.tif]

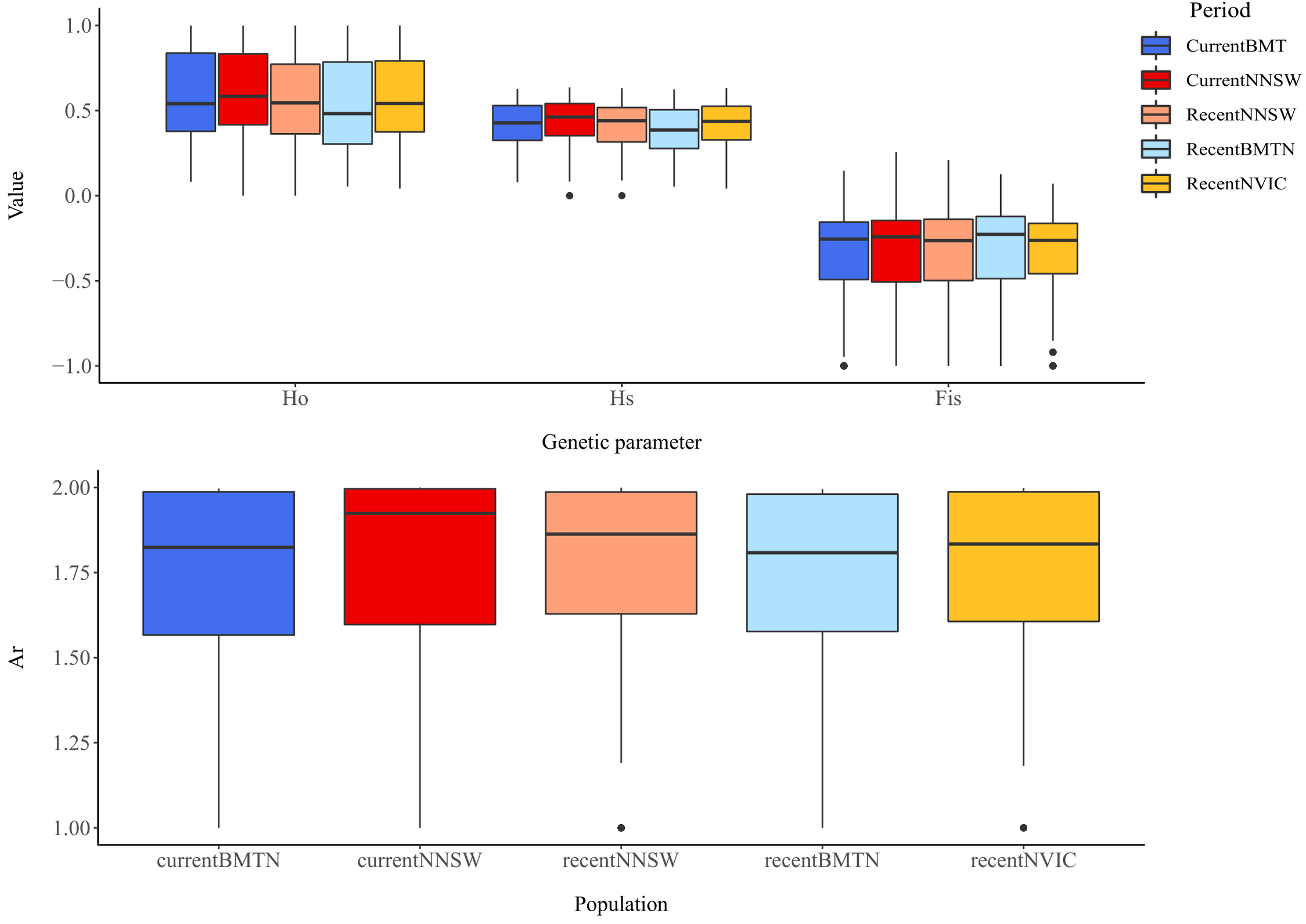

Supplement: S13 Fig — Note no current data available for N.VIC. (TIF) [file pone.0223953.s015.tif]

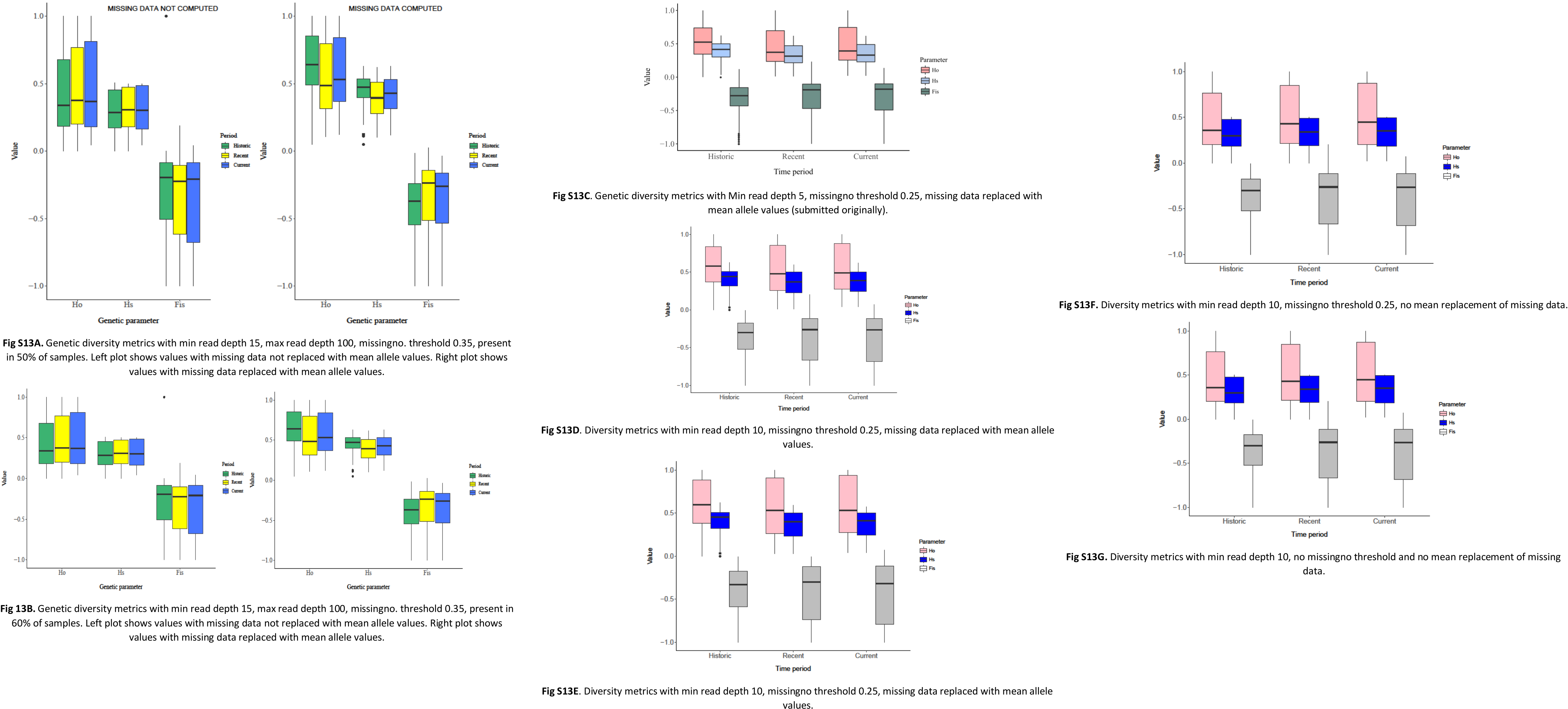

Supplement: S14 Fig — (TIFF) [file pone.0223953.s016.tiff]

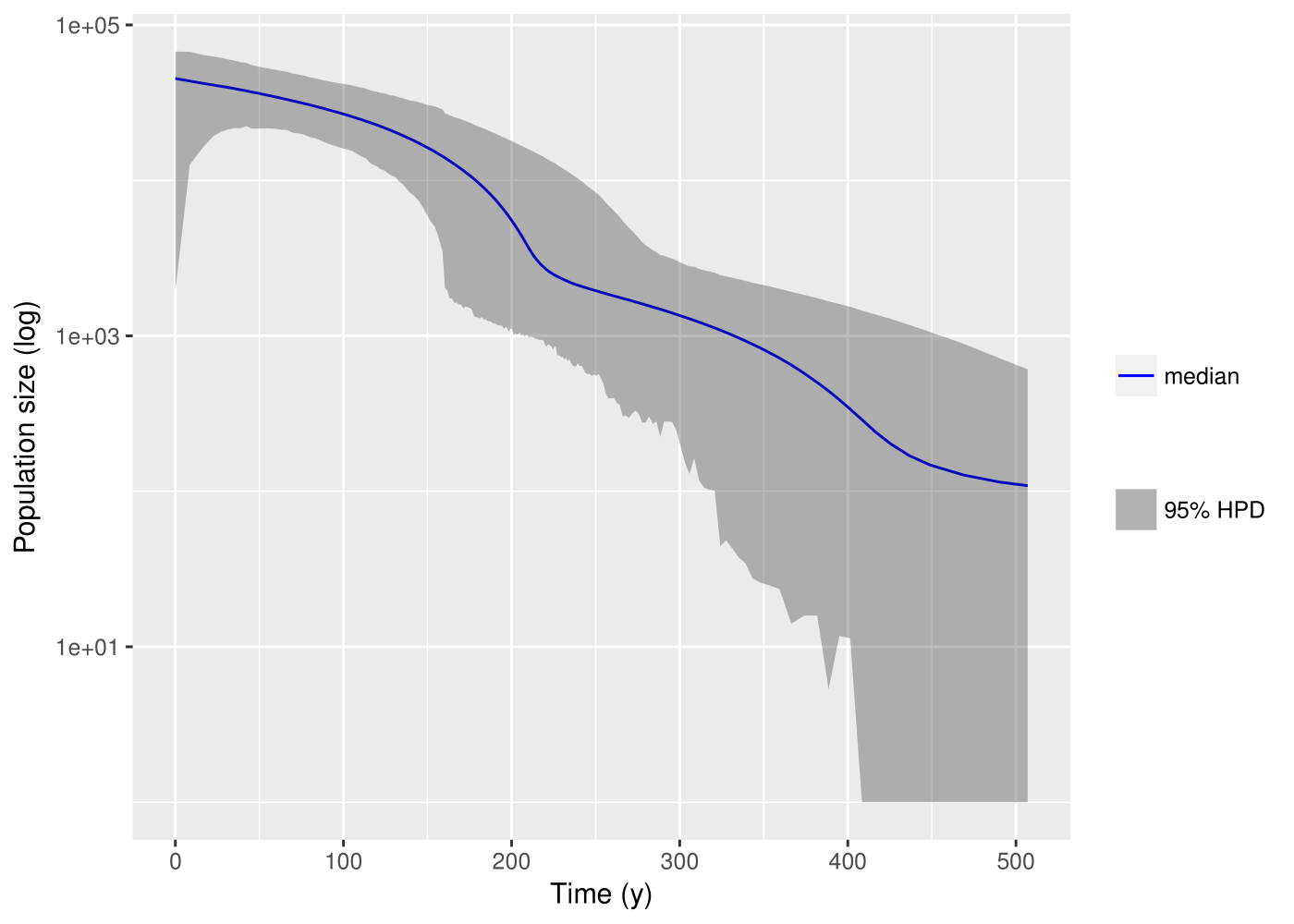

Supplement: S15 Fig — (TIF) [file pone.0223953.s017.tif]
