# Supplementary material for: Genomic impact of severe population decline in a nomadic songbird
Source: PLoS One. 2019 Oct 24;14(10):e0223953. doi: 10.1371/journal.pone.0223953 (PMC6812763; doi:10.1371/journal.pone.0223953)
Supplement: S11 Fig — Analysis of molecular variance plots of observed (black points) and simulated (grey histograms) variation within and between samples and between historic (A) and contemporary (recent and current, B) a-priori regent honeyeater populations, with FDR correction. (PDF) [file pone.0223953.s013.pdf]

Historic AMOVA:

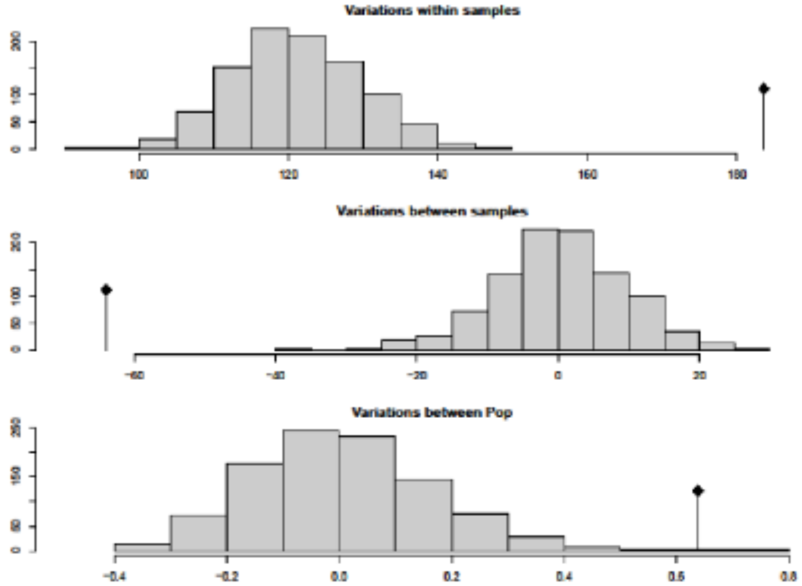

	DF	Sum Sq	Mean Sq	Sigma	% var.	Std. Obs	P_{adj}	*Phi*
Between pop	4	762.4	190.6	0.82	0.31	6.7	0.003	0.003
Between samples	148	21204.4	143.3	-120.13	-45.47	11.35	1	-0.45
Within samples	153	58682	383.5	383.5	145.15	11.95	1	-0.45
Total	305	80648.8	264.4	264.23	100			

Analysis of molecular variance plots of observed (black points) and simulated (grey histograms) variation within and between samples and between historic *a-priori* regent honeyeater populations, with FDR correction.

Contemporary AMOVA:

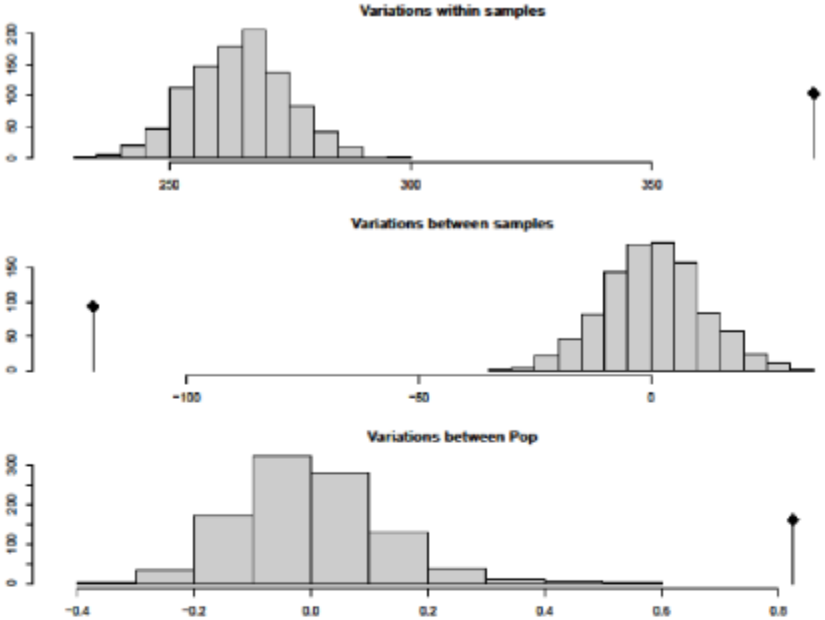

	DF	Sum Sq	Mean Sq	Sigma	% var.	Std. Obs	P_{adj}	*Phi*
Between pop	4	276.5	69.12	0.64	0.53	3.93	0.012	0.005
Between samples	53	2955.3	55.76	-63.94	-53.14	-7.03	1	-0.53
Within samples	58	10652	183.68	183.65	152.61	7.55	1	-0.53
Total	115	13883.8	120.73	120.35	100			

Analysis of molecular variance plots of observed (black points) and simulated (grey histograms) variation within and between samples and between contemporary *a-priori* regent honeyeater populations, with FDR correction.
